# Circulating tumor cell assay to non-invasively evaluate PD-L1 and other therapeutic targets in multiple cancers

**DOI:** 10.1371/journal.pone.0270139

**Published:** 2022-06-17

**Authors:** Raymond Page, Darshana Patil, Dadasaheb Akolkar, Sudha S. Murthy, Kiran Bendale, Revati Patil, Pradeep Fulmali, Pooja Fulmali, Archana Adhav, Sneha Puranik, Sachin Apurwa, Vineet Datta, Chirantan Bose, Stefan Schuster, Jinumary John, Ajay Srinivasan, Rajan Datar

**Affiliations:** 1 Department of Biochemical Engineering, Worcester Polytechnic Institute, Worcester, Massachusetts, United States of America; 2 Department of Research and Innovation, Datar Cancer Genetics, Nasik, Maharashtra, India; 3 Datar Cancer Genetics Europe GmbH, Eckersdorf, Bavaria, Germany; Institute of Biomedical Sciences, TAIWAN

## Abstract

Biomarker directed selection of targeted anti-neoplastic agents such as immune checkpoint inhibitors, small molecule inhibitors and monoclonal antibodies form an important aspect of cancer treatment. Immunohistochemistry (IHC) analysis of the tumor tissue is the method of choice to evaluate the presence of these biomarkers. However, a significant barrier to biomarker testing on tissue is the availability of an adequate amount of tissue and need for repetitive sampling due to tumor evolution. Also, tumor tissue testing is not immune to inter- and intra-tumor heterogeneity. We describe the analytical and clinical validation of a Circulating Tumor Cell (CTC) assay to accurately assess the presence of PD-L1 22C3 and PD-L1 28.8, ER, PR and HER2, from patients with solid tumors to guide the choice of suitable targeted therapies. Analytically, the test has high sensitivity, specificity, linearity and precision. Based on a blinded case control study, the clinical sensitivity and specificity for PD-L1 (22C3 and 28.8) was determined to be 90% and 100% respectively. The clinical sensitivity and specificity was 83% and 89% for ER; 80% and 94% for PR; 63% and 89% for HER2 (by ICC); and 100% and 92% for HER2 (by FISH), respectively. The performance characteristics of the test support its suitability and adaptability for routine clinical use.

## 1. Introduction

Rapid advances in the understanding of key cellular pathways that drive cancer cell survival and proliferation have guided the development and use of molecularly targeted therapies in solid tumors. These molecular targets can be cell surface receptors or particular genetic alterations such as mutations, fusions or translocations that confer an oncogenic potential to cancer cells. Such anticancer agents act on specific molecular targets expressed preferentially by neoplastic cells, and therefore are expected to be more effective with fewer side effects than conventional cytotoxic anticancer treatments (chemotherapy) [1]. A wide range of targeted drugs have been approved by the US Food and Drug Administration and also recommended as standard of care therapies for multiple solid organ tumors. Immune checkpoint inhibitors Pembrolizumab, Nivolumab, Atezolizumab, Cemiplimab-rwlc, Ipilimumab for certain PD-L1 positive tumors; hormonal therapy such as Tamoxifen, Fulvestrant, Anastrozole, Letrozole, Exemestane targeting estrogen receptor (ER) in breast cancer; anti-HER2 drugs for HER2 overexpressing breast, colon, rectal, gastric and esophageal tumors are a few examples of targeted therapies in routine clinical use.

Accurate detection of the presence of these theranostic markers in tumor samples from cancer patients is the most crucial and ongoing need for directing the choice of targeted therapy for the patient. Programmed death-ligand 1 (PD-L1) expression in tumor tissue is a predictor for the efficacy of immune checkpoint inhibitors (ICIs) in some solid tumors. In addition to limitations inherent to tissue biopsy, predicting response to ICI therapy remains a challenge owing to variation in the choice of PD-L1 detection antibodies and relevant cell population, positivity cut-off values, sample processing, spatial and temporal heterogeneity in PD-L1 expression, and oncogenic versus induced PD-L1 expression [2–4].

Biomarker-guided systemic therapy in breast cancer is based on the several molecular subtypes of the disease determined by gene expression profiling and immunohistochemistry (IHC) analysis [5–9]. Discordance in tumor characteristics, predominantly the receptor status, of primary and metastatic breast cancer is largely due to tumor progression and evolution, the choice of adjuvant therapies and sites of metastasis [10–12]. As clinically relevant discordances in hormone receptor (ER, estrogen receptor; PR, progesterone receptor) and human epidermal growth factor receptor 2 (HER2) status impact prognosis, subsequent therapy choices and patient management [11, 13–15], it warrants biopsy and retesting of the metastatic lesions for these markers [16–19]. However, biopsies of metastases or serial biopsies in case of disease progression are cumbersome, expensive, restricted to the most accessible metastatic site and do not account for intra-tumor and inter-metastatic heterogeneity. Such biopsies may also not be feasible or warranted due to poor ECOG performance score or co-morbidities.

CTC characterisation has the potential for non-invasive evaluation of these biomarkers for treatment selection as well as for longitudinal assessments to determine changes to biomarker status enabling predictive therapeutic course corrections [20–22]. We have previously demonstrated that CTCs are ubiquitous in various cancers, irrespective of radiological, metastatic or therapy status and can be utilized for non-invasive diagnostic triaging [23, 24]. Here, we demonstrate the method development, optimization, analytical and clinical validation of a CTC based technology to evaluate therapeutically relevant targets viz. PD-L1, ER, PR and HER2 from blood samples of patients with solid organ cancers to guide choice of suitable targeted therapies.

## 2. Methods

### 2.1. Samples

All biological samples for the method development, optimization and validation were obtained from participants in two studies, TRUEBLOOD (CTRI/2019/03/017918) and RESOLUTE (CTRI/2019/01/017219). Both studies, TRUEBLOOD and RESOLUTE were approved by Datar Cancer Genetics Limited Institutional Ethics Committee. The approval letters are available at:

http://ctri.nic.in/Clinicaltrials//WriteReadData/ethic/9337723928ECApprovalLetterTrueBloodAmendment.pdf

http://ctri.nic.in/Clinicaltrials//WriteReadData/ethic/6623151333ApprovalLetterbyEC-RESOLUTE.pdf

In addition, leftover blood samples from known (recently diagnosed or pre-treated) cancer patients who availed of Datar Cancer Genetics’s commercial services for cancer management as well as healthy (asymptomatic) volunteers at the organization were also obtained. All participants provided written informed consent. Both studies were performed in accordance with the Declaration of Helsinki.

Peripheral blood mononuclear cells, Formalin-fixed, Paraffin-embedded (FFPE) tissue sections, cell lines and CTCs were used for FISH. Fresh tissue collected from all study participants was transported in transport medium or RNA later solution at 4°C. FFPE tissue samples were transported to the laboratory at room temperature. All samples were processed at the DCGL Laboratory facility accredited by the College of American Pathologists (CAP) as well as Clinical Laboratory Improvement Amendments (CLIA).

### 2.2. Antisera and cell lines

The details of antisera and cell lines are provided in S1 Table. The purity of the cell lines was confirmed by Short Tandem Repeat (STR) Profiling and testing for Mycoplasma every 6 months.

### 2.3. Enrichment of circulating tumor cells from peripheral blood

Aliquoted blood samples (5 mL) were processed for the enrichment of CTCs from peripheral blood mononuclear cells (PBMC) as described previously [25]. PBMCs were treated with a proprietary differentially cytotoxic medium for up to 100 hours at 37°C under 5% CO_2_, 4% O_2_. The medium induces cell death in normal (non-malignant) cells with functional apoptotic machinery while simultaneously conferring survival privilege on apoptosis-resistant cells of tumorigenic origin, i.e. Circulating Tumor Associated Cells (C-TACs; EpCAM+, PanCK+, CD45±) and their clusters. C-TACs include CTCs (EpCAM+, PanCK+, CD45-) as well as other cell types such as Tumor Associated Macrophages (TAMs) and Tumor Associated Fibroblasts (TAFs).

### 2.4. Immunocytochemistry profiling of CTCs

The process of ICC profiling of CTCs was as described previously [24]. Representative fluorescent images of Circulating Tumor Cells (CTCs) isolated from cancer patient samples immuno-stained for A) PD-L1 22C3, B) PD-L1 28.8, C) ER, D) PR, and E) HER2 are provided in S1 Fig.

### 2.5. Fluorescence in situ hybridization (FISH)

Tissue sections were fixed on poly-L-Lysine coated slides for 1 h at 60 ^0^C and then incubated at 70 ^0^C for 10 min. Sections were dehydrated with serially diluted ethyl alcohol (increasing concentration), deparaffinized with 2 washes of xylene and finally rehydrated with serially diluted ethyl alcohol (decreasing concentration). Sections were preheated and then subjected to proteolysis with pepsin at 37 ^0^C for 35 min. Following proteolysis, sections were washed with 1x saline sodium citrate (SSC) and dehydrated with serially diluted ethyl alcohol. DNA probe was carefully overlaid over tissue sections and covered with a coverslip. Denaturation was performed at 72 ^0^C for 12 min followed by incubation in a humidified chamber for 17.5 hours. Post hybridization washing was performed twice for five minutes with wash buffer (manufacturer provided).

SKBR3 and PBMCs were diluted to ~1000 cells. CTCs with cell viability of >90% and approximately 500–1000 cells per reaction were used. Cells were fixed with fresh 3:1 methanol: glacial acetic acid for 5 min. Cells were harvested by centrifugation (400 × g, 5 min, room temperature) and resuspended in minimal diluent (as required for the number of assays). Slides were cleaned with 70% ethanol and coated with Poly-L-lysine before use. Approximately 10 μl of the fixed cell suspension was placed on the slide. Freshly prepared 3: 1 methanol–acetic acid fixative was carefully overlaid on the cell suspension where a crater forms within the cell suspension. Additional fixative was overlaid dropwise until the aqueous solution drew back to the slide edges. The slide was then drained, dried and immersed in ice-cold methanol for 5 min. Fixed cells were treated with 2x SSC for 2 min followed by enzymatic proteolysis for 15 min. Post fixation of the slides was performed using 1% formaldehyde solution for 5 min followed by washing for 15 min using 1x Tris buffered saline (TBS) and dehydration using serially diluted ethanol. DNA probe was added onto the slide and covered with a coverslip. Denaturation was performed at 72 ^0^C for 12 min followed by incubation in a humidified chamber for 17.5 hours. Post-hybridization washes (2x) were performed for 2 min with cytology stringency buffer and 5 min with cytology wash buffer SSC.

Finally, all slides (tissue / cells) were incubated with 4′,6-diamidino-2-phenylindole (DAPI) for nuclear staining. Samples were then treated with antifade mountant for 15 min at room temperature. All slides were scanned on an Axio Imager Z2 (Carl Zeiss Oberkochen, Germany). Single color images were captured in several focus planes to capture both fluorophores. The reporting of all FISH samples was as per ASCO-CAP 2018 HER2 Testing Guidelines [26–28]. Representative fluorescent images of Circulating Tumor Cells (CTCs) and corresponding tumor tissue from a breast cancer patient positive for HER2 gene amplification (green) as evaluated by FISH is provided in S2 Fig.

### 2.6. Positive and negative controls

The FISH assay included SKBR3 reference cell line as the positive control (with reported ERBB2 gene amplification) while peripheral blood mononuclear cells (PBMCs) isolated from asymptomatic individuals (males + females) with no history, suspicion or risk of cancer were used as negative control.

### 2.7. Probe design

The CE IVD approved ZytoLight SPEC ERBB2/CEN 17 Dual Color Probe (PL8) was used for fluorescence in situ hybridization. The probe is a cocktail of ZyOrange (ex: 547 nm, em: 572 nm) labelled probe specific for the alpha satellite centromeric region of chromosome 17 (D17Z1, CEN17) and ZyGreen (ex: 503 nm, em: 528 nm) labelled probe specific for the chromosomal region 17q12-q21.1 harboring the ERBB2 gene.

### 2.8. IHC analysis

FFPE tissue was analyzed by IHC using Anti-Her-2 (Polyclonal, DAKO) as per standard procedures and the results were analyzed as per ASCO-CAP guidelines.

### 2.9. Method development and optimization–ICC

**Fig 1:**
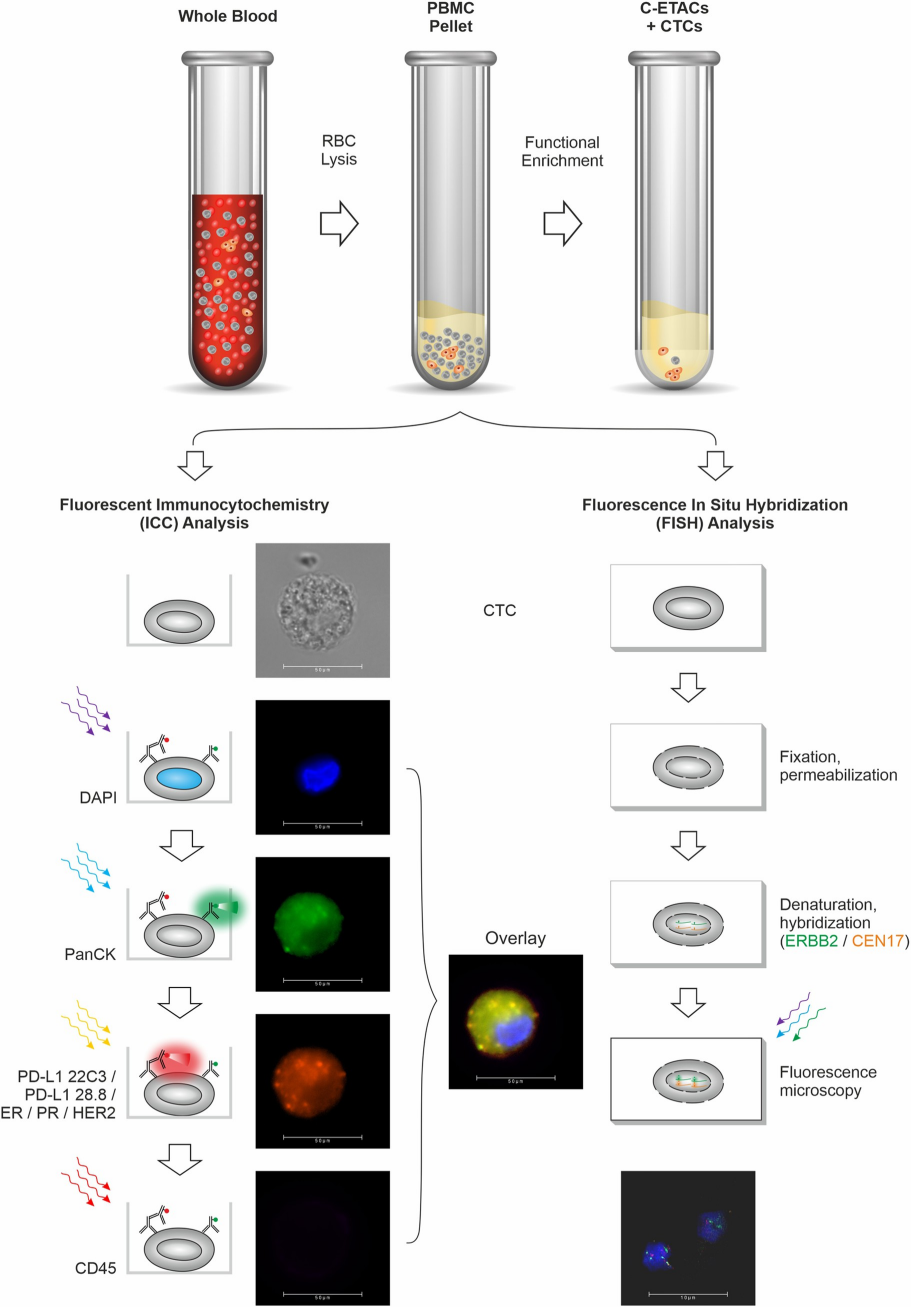
Schema of test. Functional enrichment of CTCs is achieved using an epigenetically activating medium that eliminates all non-malignant cells and permits tumor derived malignant cells to survive. Subsequently, the multiplexed immunocytochemistry (ICC) evaluates the presence of therapeutically relevant markers (PD-L1 22C3, PD-L1 28.8, ER, PR, and HER2) on respective CTCs. Fluorescence in situ hybridization (FISH) for HER2 assesses the amplification of this gene in enriched CTCs. The representative FISH image shows DAPI-stained nuclei of CTCs with HER2 gene amplification (green).

Details of method development and optimization studies as well as their findings are provided in the Supplementary materials. Fig 1 is a Schema of the test showing the various steps in CTC enrichment followed by ICC/FISH profiling for therapeutic biomarkers.

### 2.10. Analytical validation—ICC

Details of extensive analytical validations which established the performance characteristics of the test as well as their findings are provided in the Supplementary materials.

### 2.11. Analytical validation—FISH

Analytical validation of the FISH process was performed using healthy donor PBMCs and ERBB2/CEN17 positive SKBR3 reference cells. Details of the asymptomatic samples are provided in S2 Table and Supplementary materials.

### 2.12. Clinical study—ICC

The ability of the test to detect therapeutically relevant markers PD-L1, ER, PR and HER2 in the blood from cancer patients (n = 192) across 8 different solid organ tumors was ascertained and established. All clinical samples were divided into discrete non-overlapping Training and Test sets. The operator was always blinded to the clinical status of the samples (marker positive or negative) irrespective of Training or Test sets in order to eliminate any potential bias in sample processing. After generation of sample data, these were provided to the analysts who evaluated the concordance between the clinical status and the test findings. The analysts were initially unmasked to the clinical status of samples (marker positive or marker negative) in the Training set for the purpose of evaluating the initial concordance between the marker and clinical status. Concordance analysis in Training set was intended to identify markers with low or no concordance so that they may be excluded from further analysis. Once initial concordance was established, the analysts were then provided the marker status of samples in the Test set based on which the clinical status was predicted in a blinded manner. After the prediction was completed for all samples, the analysts were then unmasked to the actual clinical status of samples. The concordance between the predicted and actual clinical status was evaluated to determine the performance characteristics of the test including sensitivity, specificity and accuracy. Demographic details of the cancer patients are provided in S3 Table.

### 2.13. Clinical study—FISH

Clinical Validation of the test to detect HER2 (ERBB2) gene amplification was established across 2 studies. For the first study, a blinded case control analysis was performed on CTCs from 54 breast cancer samples where HER2 copy number status was pre-determined on corresponding tumor tissue samples by FISH. For the second study, 44 breast cancer samples were utilised to determine the concordance between FISH and IHC on tumor tissue with FISH performed on CTCs from matched blood samples of same patients.

## 3. Results

### 3.1. Statistical analysis

Statistical considerations were an integral part of method validation (analytical and clinical). In analytical validations, samples were evaluated in multiple replicates, and the mean of observations was reported along with the Coefficient of Variation (CV, %) which was determined from the Mean and Standard Deviation for Precision, Robustness, and Interference. Sensitivity, Specificity, and Accuracy were calculated using standard formulae (https://www.medcalc.org/calc/diagnostic_test.php) based on True Positives, True Negatives, False Positives, and False Negatives from testing of multiple replicates of samples and reported along with the 95% CI. Precision was derived from the Mean and Standard Deviation from testing of multiple replicates and reported as the %CV. For Linearity, the coefficient of determination was reported based on the Regression analysis performed. In clinical validation, Specificity and median values for Sensitivity and Accuracy were calculated using standard formulae based on True Positives, True Negatives, False Positives and False Negatives and the values have been reported along with the 95% CI range.

### 3.2. Analytical validation—ICC

The overall findings of the analytical validation study are presented in Table 1. The detailed findings are provided in Supplementary materials.

**Table 1:** Summary of analytical validation. The findings of Analytical Validation indicate that the Test provides reliable, accurate and reproducible results when samples are obtained, stored and processed under the recommended conditions.

	PD-L1 22C3	PD-L1 28.8	ER	PR	HER2
**Analyte Stability**	24 h	24 h	24 h	24 h	24 h
**Recovery**	>85%	>85%	>85%	>85%	>85%
**Linearity**	>0.99	>0.99	>0.99	>0.99	>0.99
**Linear Range**	7–1000 cells / 5 mL	7–1000 cells / 5 mL	5–1200 cells / 5 mL	5–1200 cells / 5 mL	5–1200 cells / 5 mL
**LoB**	0 cells / mL	0 cells / mL	0 cells / mL	0 cells / mL	0 cells / mL
**LoD**	2 cells / 5 mL	2 cells / 5 mL	3 cells / 5 mL	3 cells / 5 mL	3 cells / 5 mL
**LoQ**	7 cells / 5 mL	7 cells / 5 mL	5 cells / 5 mL	5 cells / 5 mL	5 cells / 5 mL
**Sensitivity**	87.5% (73.2% - 95.81%)	90% (76.34% - 97.21%)	87.5% (73.2% - 95.81%)	95% (83.08% - 99.39%)	92.5% (79.61% - 98.43%)
**Specificity**	100% (86.28% - 100%)	100% (86.28% - 100%)	100% (86.28% - 100%)	100% (86.28% - 100%)	100% (86.28% - 100%)
**Accuracy**	92.31% (82.95% - 97.46%)	93.85% (84.99% - 98.30%)	92.31% (82.95% - 97.46%)	96.92% (89.32% - 99.63%)	95.38% (87.10% - 99.04%)
**Precision**	CV ≤ 2.4%	CV ≤ 2.4%	CV ≤ 2.4%	CV ≤ 2.4%	CV ≤ 2.4%

### 3.3. Analytical validation—FISH

The detailed findings of the analytical validation by FISH are provided in Supplementary materials.

### 3.4. Clinical validation—ICC

We evaluated the performance characteristics of the test in a case control cross validation study. The study included sub-cohorts for PD-L1 22C3, PD-L1 28.8, ER, PR and HER2 where status of markers (positive or negative) was previously established via immunohistochemistry (IHC) of tumor tissue obtained by invasive biopsy. Samples were assigned to training and test sets in a 70%:30% ratio. Following initial assignment of samples into training and test sets, samples were shuffled and a random 30% selected successively to generate a total of 20 iterations of the test set. The performance characteristics of the test were evaluated across the 20 iterations of the test set (S4 Table); this cross validation model avoided anomalous results from overfitting (biased selection) as well as random enrichment (unbiased selection) of samples in the test set. Based on the cross validation, the median sensitivities for the markers as well as the best and worst case scenarios are presented in Table 2. The median sensitivity was 90% for PD-L1 22C3, 90% for PD-L1 28.8, 83% for ER, 80% for PR and 63% for HER2. The median specificity was 100% for PD-L1 22C3, 100% for PD-L1 28.8, 89% for ER, 94% for PR and 89% for HER2.

**Table 2:** Clinical performance characteristics. The table provides the performance characteristics of the Test which were determined from 20 iterations of the Test Set and the Best-, Median- and Worst-Case values are reported.

		Sensitivity	Specificity	Accuracy
**PD-L1 22C3**	**Training Set**	86%	96%	92%
	**Test Set (Worst)**	70% (50% - 90%)	100% (100% - 100%)	86% (71% - 100%)
	**Test Set (Median)**	90% (77% - 100%)	100% (100% - 100%)	95% (86% - 100%)
	**Test Set (Best)**	100% (100% - 100%)	100% (100% - 100%)	100% (100% - 100%)
**PD-L1 28.8**	**Training Set**	86%	96%	92%
	**Test Set (Worst)**	70% (50% - 90%)	91% (79% - 100%)	86% (71% - 100%)
	**Test Set (Median)**	90% (77% - 100%)	100% (100% - 100%)	90% (78% - 100%)
	**Test Set**	100%	100%	100%
	**(Best)**	(100% - 100%)	(100% - 100%)	(100% - 100%)
**ER**	**Training**	83%	94%	88%
	**Set**			
	**Test Set**	72%	71%	78%
	**(Worst)**	(57% - 88%)	(56% - 87%)	(64% - 92%)
	**Test Set (Median)**	83%	89%	84%
		(70% - 96%)	(79% - 100%)	(72% - 97%)
	**Test Set**	94%	100%	94%
	**(Best)**	(87% - 100%)	(100% - 100%)	(85% - 100%)
**PR**	**Training**	83%	92%	88%
	**Set**			
	**Test Set**	60%	88%	77%
	**(Worst)**	(43% - 77%)	(76% - 99%)	(63% - 92%)
	**Test Set**	80%	94%	87%
	**(Median)**	(66% - 94%)	(85% - 100%)	(75% - 99%)
	**Test Set**	93%	100%	97%
	**(Best)**	(85% - 100%)	(100% - 100%)	(91% - 100%)
**HER2**	**Training**	67%	91%	84%
	**Set**			
	**Test Set**	38%	72%	69%
	**(Worst)**	(19% - 56%)	(55% - 89%)	(51% - 87%)
	**Test Set (Median)**	63%	89%	79%
		(44% - 81%)	(77% - 100%)	(63% - 95%)
	**Test Set**	100%	100%	92%
	**(Best)**	(100% - 100%)	(100% - 100%)	(82% - 100%)
**HER2 (FISH)**	**Training**	89%	97%	95%
	**Set**			
	**Test Set**	75%	83%	81%
	**(Worst)**	(54% - 96%)	(65% - 100%)	(62% - 100%)
	**Test Set**	100%	92%	94%
	**(Median)**	(100% - 100%)	(78% - 100%)	(82% - 100%)
	**Test Set**	100%	100%	100%
	**(Best)**	(100% - 100%)	(100% - 100%)	(100% - 100%)

### 3.5. Clinical validation—FISH

Clinical Validation of the test was established across 2 studies.

#### 3.5.1. Study 1

The first study was a blinded case control study using CTCs from 54 known cases of breast cancer where ERBB2 gain (or its absence) was previously established on biopsied tumor tissue samples by FISH. This study employed a 20-fold cross validation design to determine the sensitivity and the specificity.

Based on the cross validation, the median sensitivities for the markers as well as the best and worst case scenarios are presented in Table 2 and S4 Table (HER2 FISH). The median sensitivity, specificity and accuracy was 100%, 92% and 94% for ERBB2 on CTCs as evaluated by FISH.

#### 3.5.2. Study 2

The second study was based on samples from 44 known cases of breast cancer. The samples in this study included a subset of samples from the first study as well as additional clinical samples which were not included in the first study. In the samples included in this study ERBB2 (HER2) status was previously established on tumor tissue by immunohistochemistry (IHC) as well as by FISH and on CTCs from matched blood samples collected from the same patients. The second study included samples where IHC of tumor tissue had indicated equivocal findings for HER2. This study established the concordance of tumor tissue-FISH as well as CTC-FISH with tumor tissue-IHC.

Among the 44 malignant samples where C-ETACs were analyzed, IHC indicated HER2 positivity in 10 samples, negative status in 25 samples and equivocal status in 9 samples. Among the HER2 positive samples, tissue FISH and CTC FISH had sensitivities of 80% and 90% respectively. Among the HER2 negative samples tissue FISH and CTC FISH had specificities of 100% and 96% respectively. Among the equivocal samples both tissue FISH and CTC fish indicated positive status in the same 2 samples and negative status in the same 7 samples (S5 Table).

## 4. Discussion

We describe a non-invasive CTC-based assay for evaluation of therapeutically relevant biomarkers PD-L1, ER, PR and HER2 in blood samples from cancer patients with potential for clinical application in solid organ tumors. This test will be especially beneficial in cases where tissue insufficiency is encountered and / or an invasive tissue biopsy for tumor profiling is unviable. The test showed high analytical as well as clinical performance characteristics, which support its intended use for evaluation of target biomarkers for therapy selection.

Target biomarker expression is a prerequisite to identify cancer patients who are likely to benefit from targeted anticancer agents such as small molecules (e.g., TKI) or monoclonal antibodies (e.g., immune checkpoint inhibitors (ICI)). Although tumor tissue analysis by IHC remains the gold standard for most clinical molecular analysis for targeted therapy selection (with the exception of NGS for EGFR, BRAF or NTRK gene variants), it faces several biological and technological challenges. Liquid biopsy can be a viable alternative to tissue biopsy for determining expression of target biomarkers to guide therapy selection. Circulating tumor cells (CTCs), being the cells of tumorigenic origin in the bloodstream, represent an ideal biomarker for theranostic applications in cancer therapy [21]. Serial monitoring of tumor marker profile for monitoring dynamic alterations in marker status during disease progression or treatment is viable with CTCs which is not possible with tumor tissue. Changes in biomarker status have been previously reported in up to 42% of cases evaluated for either PD-L1, ER, PR or HER2 [29–35].

We have earlier demonstrated the clinical utility of circulating tumor cells enriched by this unique method, for screening, diagnostic triaging and non-invasive assessment of response to cytotoxic chemotherapy agents (CCA) in solid organ cancers [23–25]. In this study, we establish the clinical utility of CTCs obtained from patients with solid tumors in evaluating the therapeutically relevant targets, PD-L1, ER, PR and HER2 to guide choice of targeted therapies.

Targeting the immune checkpoint proteins (PD-L1 or PD-1) with inhibitory mABs is a treatment strategy in multiple cancers. The expression of PD-1 and PD-L1 proteins, is considered to be one of the factors predictive of response to ICI. IHC profiling of PD-L1 status is routinely used to identify patients likely to benefit from ICI therapies. Despite the reported value of assessing PD-L1 overexpression on cells of different types in solid tumors as a promising marker, its predictive value is restricted due to limitations of tumor tissue biopsy, dynamic expression profile of PD-L1, intratumoral heterogeneity as well as the influence of immune cell infiltrate in the tumor and its microenvironment [2, 36]. Here, we demonstrate high specificity (100%) and sensitivity (90%) for both PD-L1 22C3 and PD-L1 28.8 antibody clones for detecting PD-L1 positive CTCs in patient samples. Although, the recommendation for ICI therapy based on our assay will be for cancer types as per standard guidelines and recommendations, owing to the nature of the analyte, we could eventually extend it to a tumor-agnostic setting.

Cheng et al. recently demonstrated the feasibility of CTC PD-L1 detection in peripheral blood using membrane filtration based on size [37]. Another study presented that PD-L1 status in CTCs and circulating WBCs correlate with PD-L1 status in tumor tissue, revealing the potential of CTCs assessment as a non-invasive real-time biopsy to evaluate PD-L1 expression in patients with advanced-stage NSCLC [38]. Bergmann et al also evaluated the feasibility to detect CTC-PD-L1 expression in patients with advanced urothelial carcinoma using the CellSearch® system [39]. Although these studies have shown the feasibility and prognostic value of PD-L1 expression on CTCs, none of them have explored their therapeutic utility. A recent study by Choi et al on circulating tumor cell proportion scoring (CTPS) based PD-L1 assessment concluded that pure-CTCs based CTPS could be deployed for innovative diagnosis strategies as alternatives for tissue biopsy and to guide the personalized treatment in NSCLCs [40].

ER, PR and HER2 status is prognostic and predictive in breast cancer [41, 42]. The choice of targeted therapies in breast, ovarian and uterine neoplasms depends on the expression of ER/PR on the tumor cells. Aromatase inhibitors Anastrozole, Letrozole, Exemestane; Estrogen receptor antagonists Tamoxifen and Fulvestrant are indicated for use in ER positive breast cancer. Aromatase inhibitors, Megestrol acetate, Medroxyprogesterone acetate and GnRH analogs are recommended for use in ER/PR-positive uterine sarcomas. HER2 positivity is associated with clinical benefit from anti-HER2 therapies in breast, colon, rectal, esophageal and gastric cancers. With our test, we show sensitivity of 83%, 80% and 63% and specificity of 89%, 94%, 89% for ER, PR, HER2 respectively on CTCs derived from known cancer patients. Studies have reported significant heterogeneity between ER/PR/HER2 protein expression in CTCs and primary tumor/metastatic biopsy, and this status may change over time due to therapy [11, 42–45]. In our CTC-based test, we observed slightly lower sensitivity for HER2 by ICC than other markers. However, we observed 100% median sensitivity and 92% specificity for HER2 as determined by FISH. Further, CTC FISH had sensitivity of 90% among HER2 positive samples, and 96% specificity among HER2 negative samples with 100% concordance with tissue-FISH for equivocal samples (Table 2 and S5 Table).

The extent of discordance in marker status is varied across studies probably owing to different techniques used for evaluation of marker status. Clonal selection of minor subtype of cells “hidden” within the primary tumor following treatment, tumor heterogeneity, CTC selection process or inaccurate receptor status assessment of the primary tumor are speculated to be contributing factors for the observed discordance in tumor marker profile of primary tumor and CTCs [46, 47]. A few studies elucidating the clinical relevance of this observation have been carried out. In the Treat CTC randomized phase II trial, patients with HER2 non-amplified breast cancer and ≥1 centrally confirmed CTC/15 ml of blood were randomized (1:1) to Trastuzumab treatment. Trastuzumab did not decrease the detection rate of CTCs in HER2 non-amplified, non-metastatic breast cancer. However, as patients were eligible for this trial irrespective of CTCs HER2 status, the conclusions of this trial may not be directly relevant here [48]. The results of the randomized DETECT III trial suggested that Lapatinib resulted in early declines in circulating tumor cell counts (CTCs) in patients with initially HER2-negative metastatic breast cancer but HER2-positive CTCs [49]. Thus, it seems imperative to evaluate CTC based tumor marker status for personalised therapy guidance and monitoring.

Recent studies have described the evaluation of biomarkers—PD-L1, ER, PR and HER2 on CTCs and have emphasized on the clinical utility of CTCs to guide therapy selection [50–53]. While these approaches provide initial proof of concept for CTC-based biomarker analysis, their clinical application has not yet been achieved due to low efficiency of CTC capture techniques in these studies. Our test uses a proprietary CTC enrichment method which yields sufficient CTCs in almost all samples (>95%) for meaningful downstream applications such as the evaluation of these biomarkers for therapy selection. This is reflected in the higher clinical sensitivity and overall concordance of our test. Therefore, this test is a far superior alternative for CTC-based evaluation of these therapeutically relevant biomarkers and has immediate clinical application.

The test has not been presently validated for concurrent analysis of multiple markers within a single multiplexed analysis. The test shows a superior sensitivity (100%) for evaluation of HER2 on CTCs using FISH but the same high accuracy was not observed for evaluation of HER2 by ICC. Discordances in HER2 assessment based on protein expression (ICC/IHC) and gene amplification (FISH) have been reported in literature. HER2 gene amplification seems to be very constant in different tumor regions, as opposed to greater variability observed with protein expression as assessed by IHC staining. This in turn is reflected in lower concordance rates between IHC and FISH [54–56]. As HER2-ICC also evaluates HER2 protein expression similar to IHC, it is subject to this observed variability, which may be the cause of decreased sensitivity as compared to HER2-FISH in our assay.

Presently, the test is qualitative in nature because although the test determines positivity based on the presence of CTCs above a numerical threshold, the output is binary, i.e., (Positive or Negative). Since the test is not intended to provide quantitative insight into biomarker expression such as the magnitude of antigen expression or the proportion / number of marker positive cells, it is also not intended as a surrogate marker of treatment outcomes or disease status. Furthermore, this test has not been prospectively evaluated for treatment response and patient survival where CTC profiling-based treatment guidance was used for therapy selection.

Overall, the study findings indicate that our assay for ICC characterisation of CTCs can substitute IHC analysis of tumor tissue for profiling of therapeutically relevant markers. This approach has application in cases where tumor tissue may be limited and / or where an invasive biopsy to obtain tumor tissue may be unviable as also where tumor evolution is suspected.

## 5. Conclusions

We describe a blood-based, non-invasive test which detects therapeutic biomarkers on CTCs with high sensitivity and specificity. The CTC based detection of PD-L1, ER, PR and HER2 markers offers a non-invasive alternative to tissue-based IHC for selection of immune-checkpoint inhibitors and targeted therapies for treatment of solid tumors. The test has the potential to accommodate spatial and temporal heterogeneity and is ideal for repeat sampling, longitudinal monitoring of tumor evolution and predictive therapeutic course corrections.

## Supporting information

S1 Fig Fluorescence images (ICC) of CTCs. (DOCX)

S2 Fig Fluorescence images (FISH) of CTCs and corresponding tumor tissue. (DOCX)

S3 Fig Fluorescence Intensities of markers singly and in multiplexed combinations. (DOCX)

S4 Fig Detection thresholds. (DOCX)

S5 Fig Analytical validation: Linearity (ICC and HER2-FISH). (DOCX)

S1 Table Antisera and control cells. (DOCX)

S2 Table Details of asymptomatic samples (HER2-FISH analysis). (DOCX)

S3 Table Demographics of clinical validation cohort (ICC and HER2-FISH). (DOCX)

S4 Table Cross validation. (DOCX)

S5 Table Clinical validation (HER2 FISH Cohort 2)—three-way concordance study. (DOCX)

S6 Table Markers, fluorophores and detection. (DOCX)

S7 Table Optimization of antibody dilution. (DOCX)

S8 Table Analytical validation: Linearity. (DOCX)

S9 Table Analytical Validation: Analyte stability and recovery (spiked cells). (DOCX)

S10 Table Analytical validation: Analyte stability and recovery (CTCs). (DOCX)

S11 Table Analytical validation: Sensitivity, specificity, accuracy. (DOCX)

S12 Table Analytical validation: Precision. (DOCX)

S13 Table Hybridization efficiency (HER2-FISH analysis). (DOCX)

S14 Table Precision (HER2-FISH analysis). (DOCX)

S15 Table Analytical sensitivity (HER2-FISH analysis). (DOCX)

S16 Table Analytical specificity (HER2-FISH analysis). (DOCX)
