# Supplementary material for: Circulating tumor cell assay to non-invasively evaluate PD-L1 and other therapeutic targets in multiple cancers
Source: PLoS One. 2022 Jun 17;17(6):e0270139. doi: 10.1371/journal.pone.0270139 (PMC9205490; doi:10.1371/journal.pone.0270139)
Supplement: S1 Fig — (DOCX) [file pone.0270139.s001.docx]

**S1 Fig. Fluorescence images (ICC) of CTCs**

Representative fluorescent images of Circulating Tumor Cells (CTCs) from cancer patient samples immuno-stained for A) PD-L1 22C3, B) PD-L1 28.8, C) ER, D) PR, E) HER2.

**
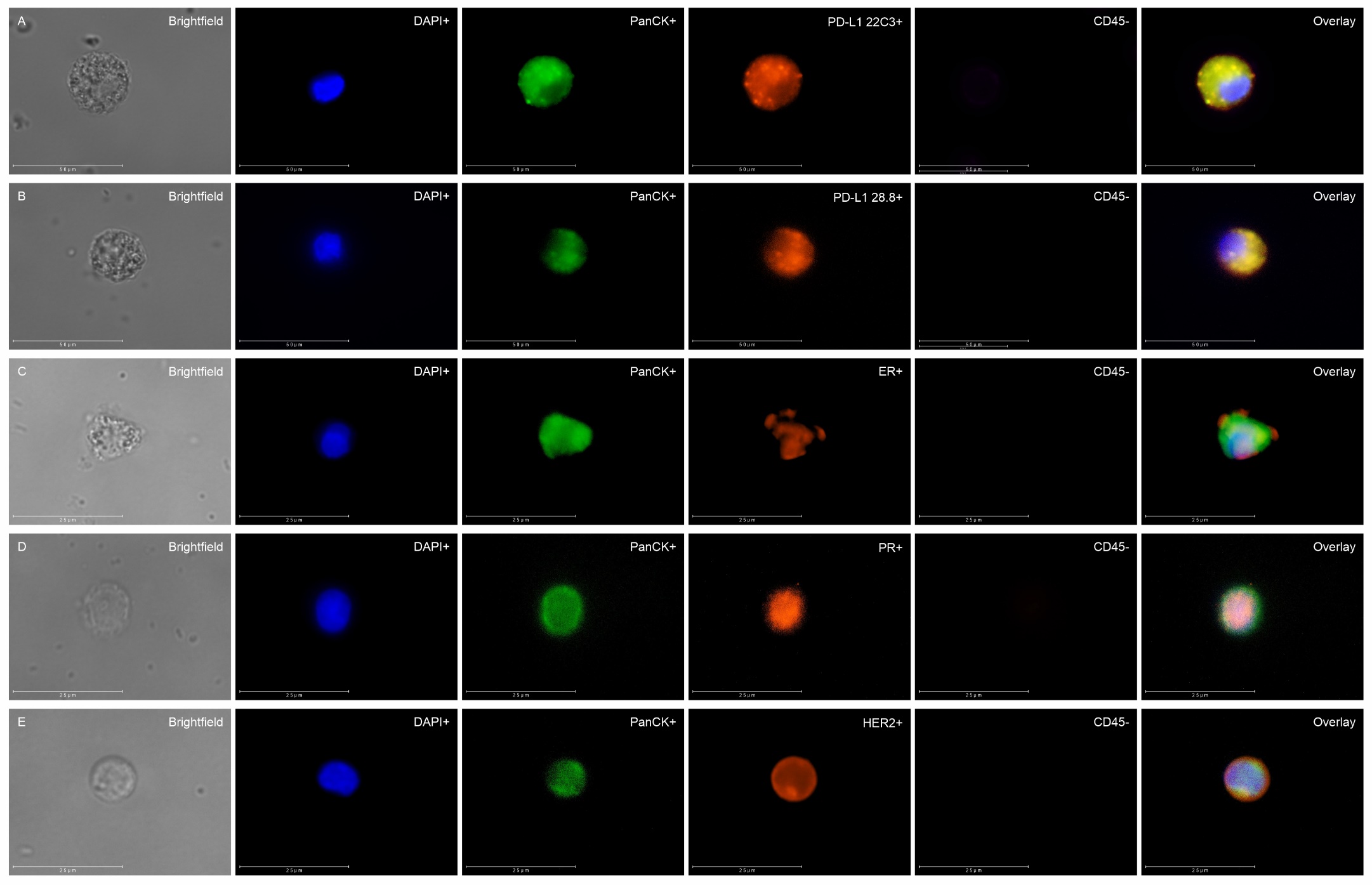
**
