# Supplementary material for: Circulating tumor cell assay to non-invasively evaluate PD-L1 and other therapeutic targets in multiple cancers
Source: PLoS One. 2022 Jun 17;17(6):e0270139. doi: 10.1371/journal.pone.0270139 (PMC9205490; doi:10.1371/journal.pone.0270139)
Supplement: S2 Fig — (DOCX) [file pone.0270139.s002.docx]

**S2 Fig. Fluorescence images (FISH) of CTCs and corresponding tumor tissue**

Representative fluorescent images of DAPI-stained nuclei of Circulating Tumor Cells (CTCs) and corresponding tumor tissue from a breast cancer patient positive for HER2 gene amplification (green) as evaluated by FISH.

**
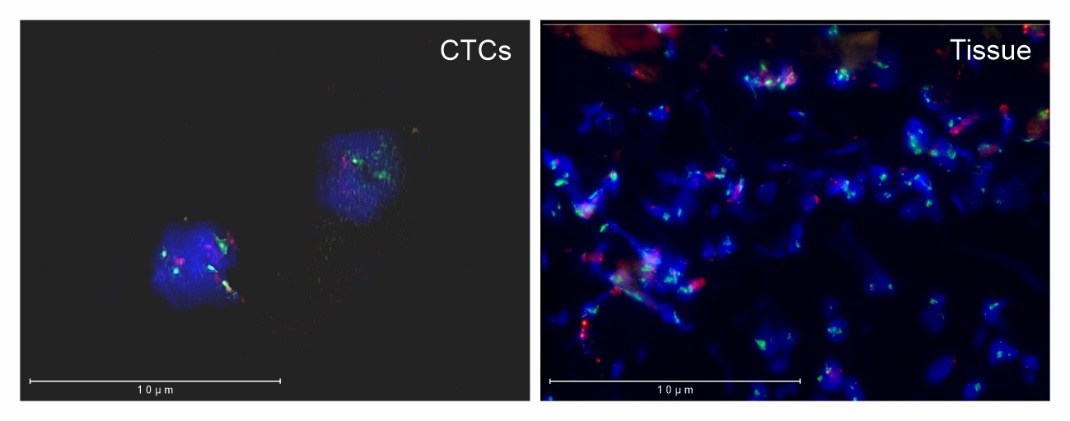
**
