# Supplementary material for: Circulating tumor cell assay to non-invasively evaluate PD-L1 and other therapeutic targets in multiple cancers
Source: PLoS One. 2022 Jun 17;17(6):e0270139. doi: 10.1371/journal.pone.0270139 (PMC9205490; doi:10.1371/journal.pone.0270139)

**Method Development and Optimization**

*Antibody Multiplexing*

Respective reference cells were immunostained with each Ab singly or in combination with other Ab, with 6 replicates per combination. The FI for each marker was evaluated for differences between single-Ab wells and in wells where Ab were used in combinations.

Use of Ab in multiplexed combinations was not associated with any significant suppression in FI of any of the markers (which may have led to loss of sensitivity). The findings established that the Ab were conducive to multiplexed analysis without any interfering effects (S3 Fig).

**S3 Fig. Fluorescence Intensities of Markers Singly and in Multiplexed Combinations**.

There was no significant suppression or elevation of the FI of markers when the fluorophore conjugated Ab were used singly or in multiplexed combinations.


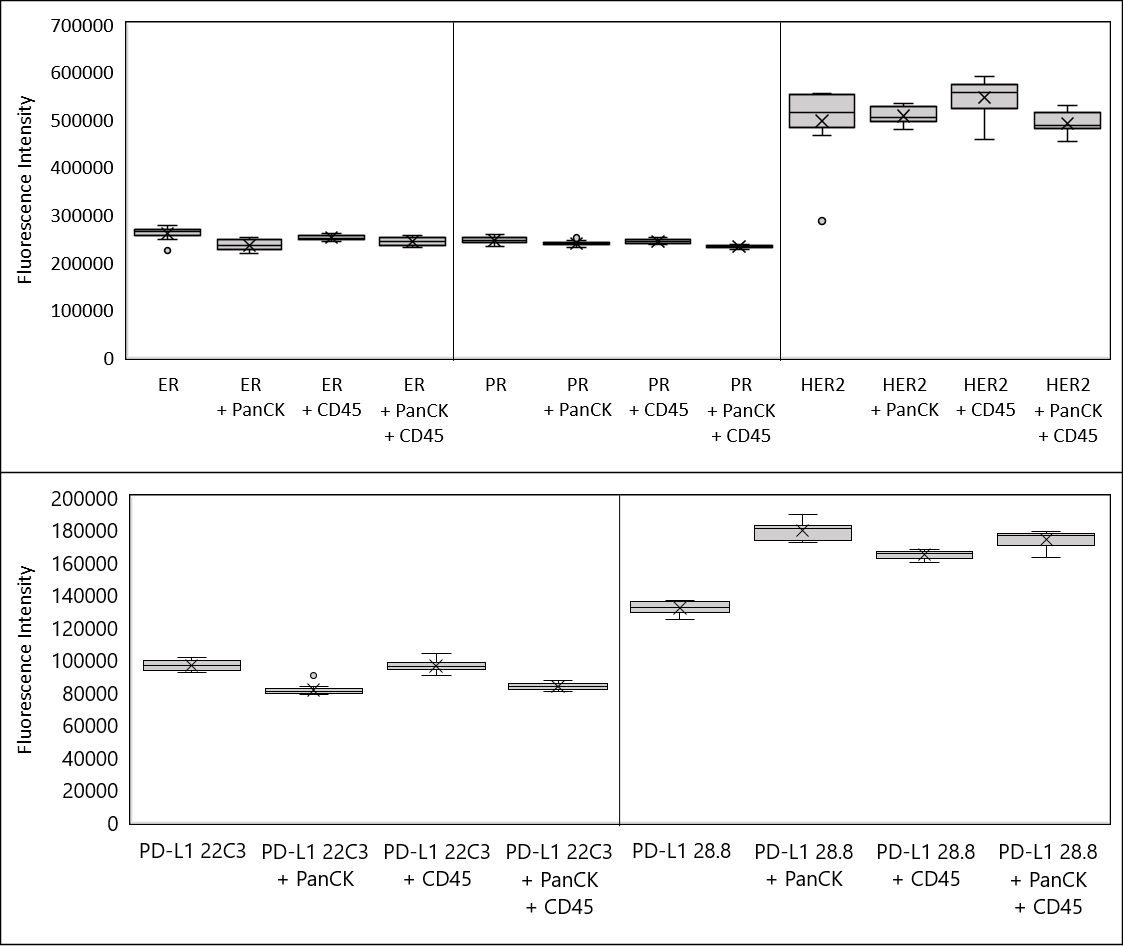

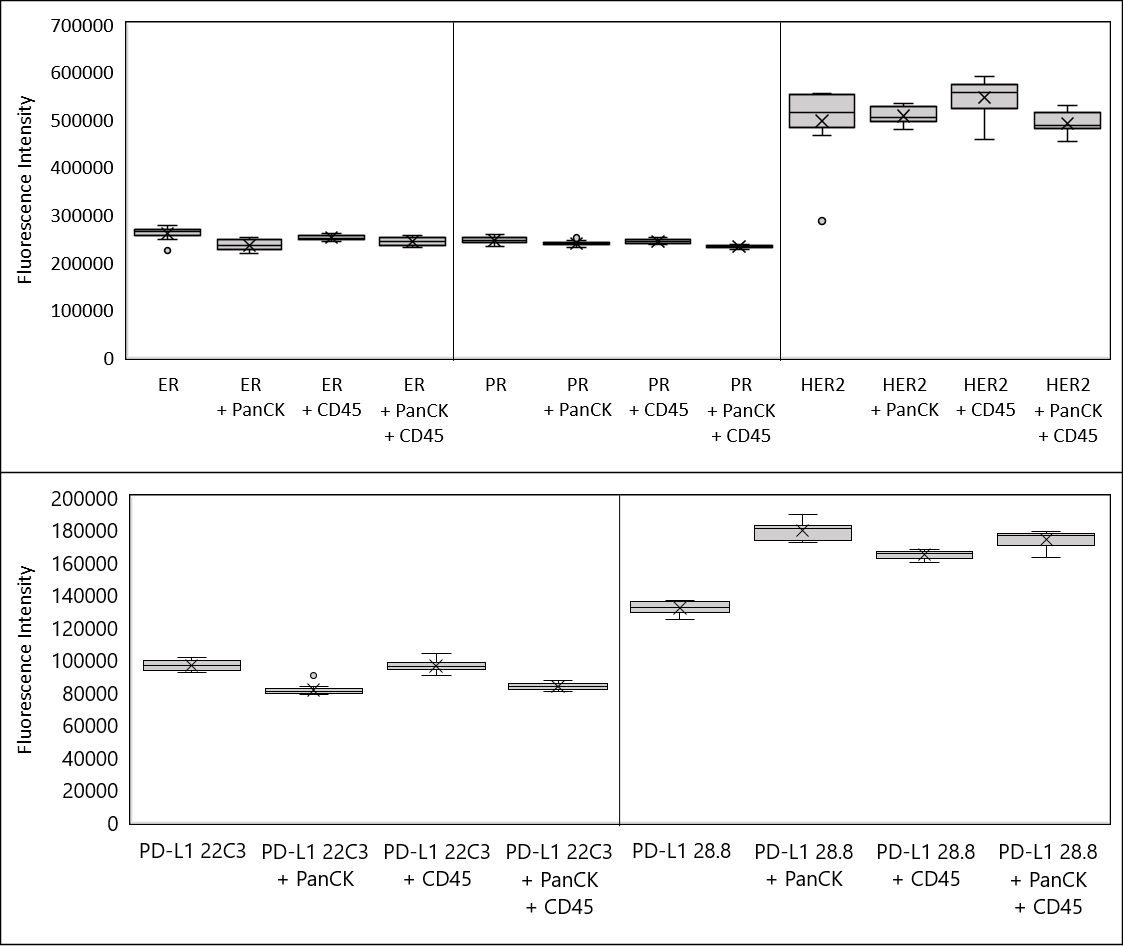

Supplement: S3 Fig — (DOCX) [file pone.0270139.s003.docx]
