# Supplementary material for: Circulating tumor cell assay to non-invasively evaluate PD-L1 and other therapeutic targets in multiple cancers
Source: PLoS One. 2022 Jun 17;17(6):e0270139. doi: 10.1371/journal.pone.0270139 (PMC9205490; doi:10.1371/journal.pone.0270139)

**Method Development and Optimization**


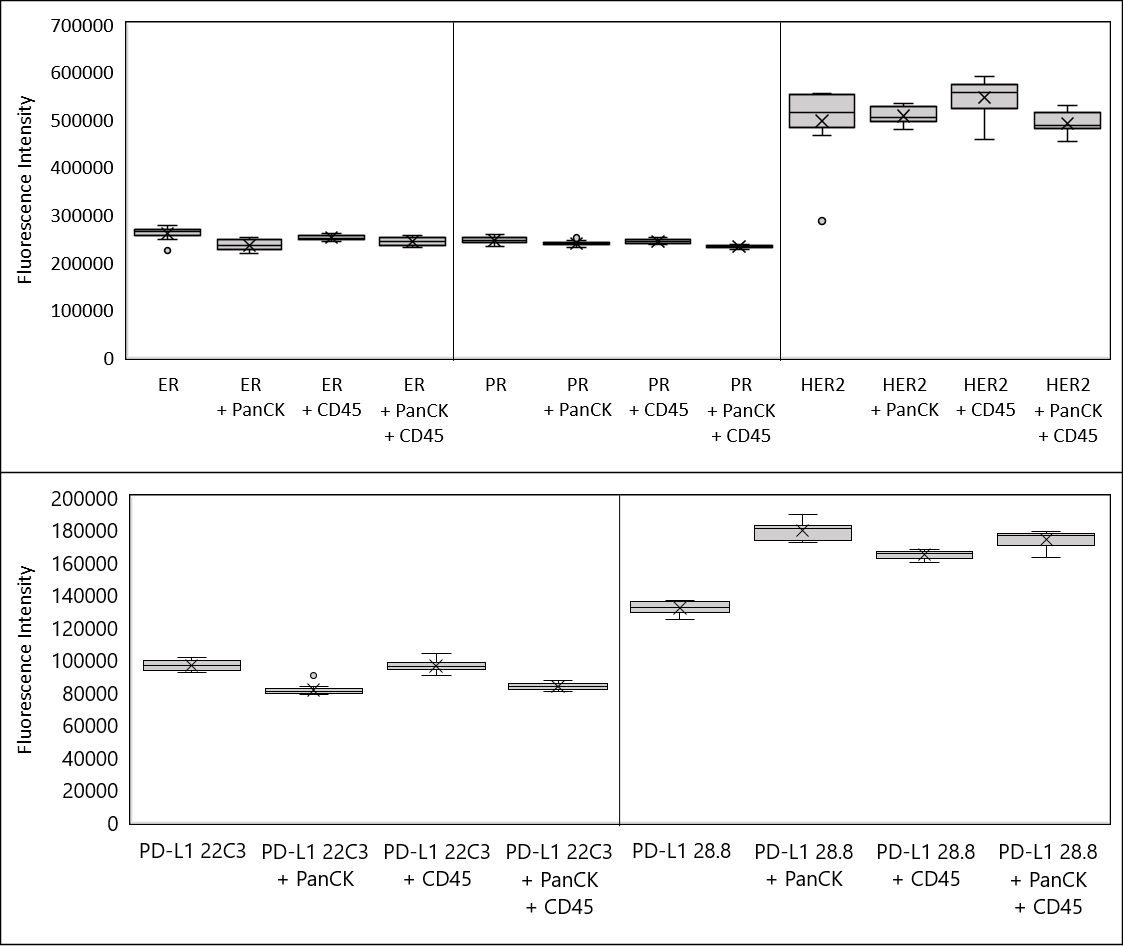


*Detection Thresholds*

Reference cell line MDA-MB-231, known PD-L1 negative (N) and positive (P) cells from a primary malignant breast tumor (TDCs), cells from a benign breast tumor (B-TDCs) and cells from a healthy (asymptomatic) individual were immunostained to determine the expression (FI) of PD-L1 22C3 and PD-L1 28.8. The FI of PD-L1 22C3 and PD-L1 28.8 was higher in MDA-MB-231 and P-TDCs than B-TDCs and N-TDCs. Reference cell lines, triple (ER, PR, HER2 markers) negative (TN) and triple positive (TP) cells from a primary malignant breast tumor (TDCs), cells from a benign breast tumor (B-TDCs) and cells from a healthy (asymptomatic) individual were immunostained to determine the expression (FI) of ER, PR and HER2. The FI of ER and PR was higher in MCF7 and TP-TDCs, while FI of HER2 was higher in SKBR3 and TP-TDCs than MDA-MB-231, B-TDCs and TN-TDCs. The FI of PanCK was higher in SKBR3, primary malignant breast tumor cells (M-TDC) and Breast adenocarcinoma specific Circulating Tumor Cells (BrAD-CTCs) than in SW982 and benign breast tumor cells (B-TDC) (S4 Fig).

Based on these findings, the FI threshold for positivity was assigned as 50,000 (relative fluorescence units, RFU) for PD-L1 22C3, ER and PR; 60,000 RFU for PD-L1 28.8 and HER2; and 70,000 RFU for PanCK. These apply as a lower threshold where expression is essential for positivity, but also accommodate CTCs with lower marker expression than the reference cell lines.

**S4 Fig. Detection Thresholds**.

The expression level of each marker was evaluated on reference cell lines, malignant breast tumor derived cells (TDCs), benign breast tumor derived cells (B-TDC) and Breast adenocarcinoma specific Circulating Tumor Cells (BrAD-CTCs). The expression levels were considered while assigning positivity thresholds for each marker.


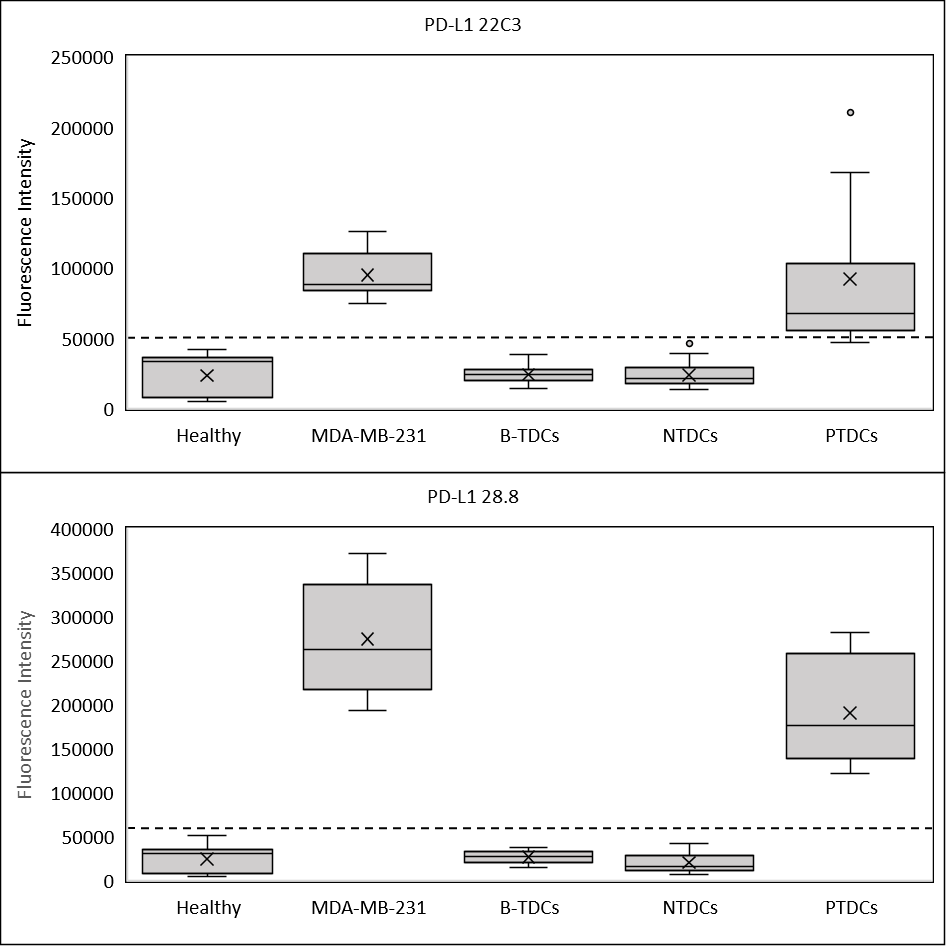

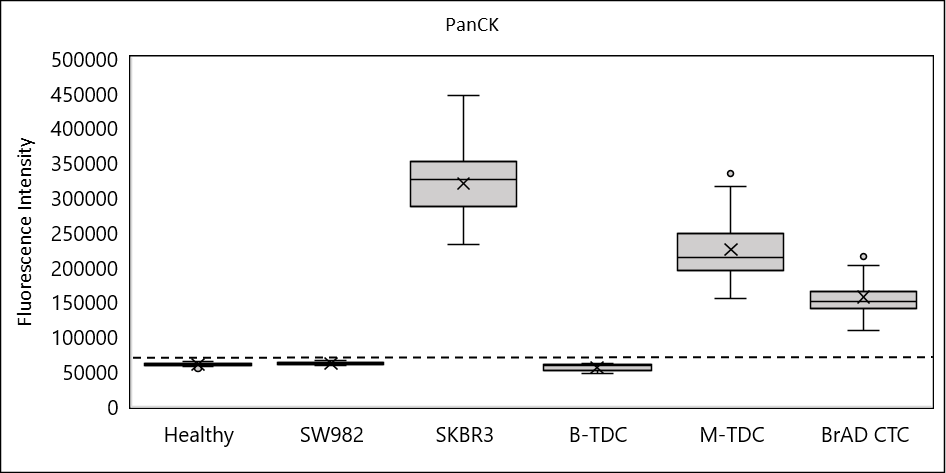


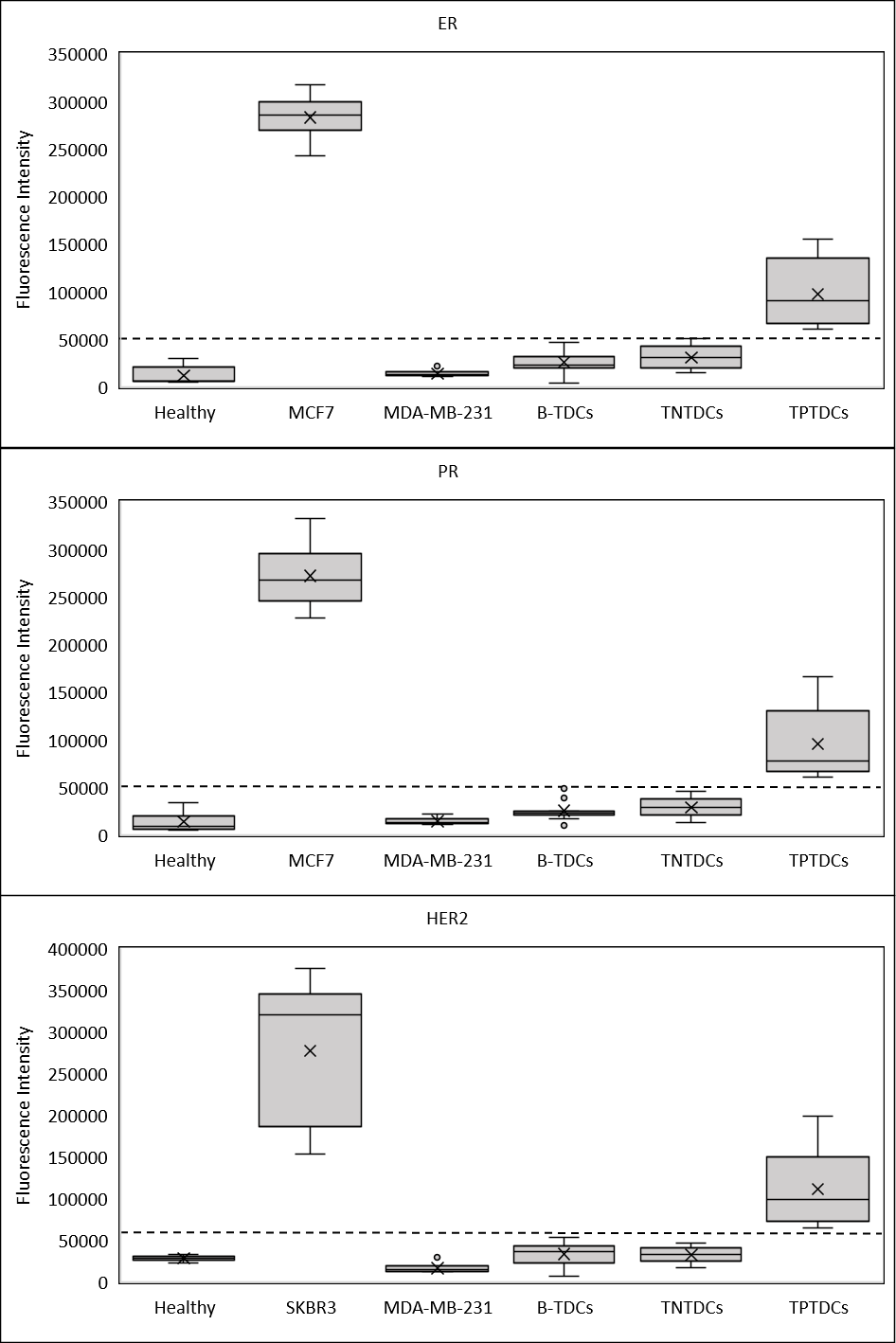

Supplement: S4 Fig — (DOCX) [file pone.0270139.s004.docx]
