# Supplementary material for: Circulating tumor cell assay to non-invasively evaluate PD-L1 and other therapeutic targets in multiple cancers
Source: PLoS One. 2022 Jun 17;17(6):e0270139. doi: 10.1371/journal.pone.0270139 (PMC9205490; doi:10.1371/journal.pone.0270139)
Supplement: S5 Fig — (DOCX) [file pone.0270139.s005.docx]

**Analytical Validation - ICC**

*Linearity*

Recoveries of spiked cells were generally higher at higher spike densities (8 cells / 5 ml for PD-L1 22C3 and 28.8; 7 cells/ 5 ml for ER, PR, HER2) (S5 Fig, S8 Table). R^2^ ≥0.99 in all markers indicated a significant linear response, especially in the range of 7 – 1000 cells / 5 mL for PD-L1 and 7 – 1200 cells / 5 mL for ER, PR and HER2.

*Limits of Detection, Quantitation and Blank*

The Limit of Blank (LoB) was defined as the background detection rate of marker positive cells by the test in absence of the reference cells. LoB was determined from the unspiked healthy donor blood samples in the Linearity Study.

The Limit of Detection (LoD) was defined as the lowest count of reference cells that could be detected in spiked samples and reliably distinguished from unspiked (blank) samples by the test, irrespective of the mean recovery among the multiple replicates tested at each spike density. The LoD was determined from the samples in the Linearity Study which had been spiked with the three lowest values of reference analyte.

The Limit of Quantitation (LoQ) was defined as the lowest count of reference cells that could be detected in spiked samples with a mean recovery of ≥80% across the multiple replicates tested at each spike density and reliably distinguished from unspiked (blank) samples by the test. The LoQ was determined from the samples in the Linearity Study which had been spiked with the five lowest values of reference analyte.

No PD-L1 22C3+, PD-L1 28.8+, ER+, PR+ or HER2+ cells were detected in the unspiked samples, i.e., no false positives. Thus, the limit of blank (LoB) was determined to be 0 cells / mL. The Limit of Detection (LoD) was 2 cells/ 5ml for PD-L1 22C3 and PD-L1 28.8; and 3 cells/ 5 ml for ER, PR and HER2. The Limit of Quantitation (LoQ) was 7 cells / 5 mL for PD-L1 22C3 and 28.8 and 5 cells / 5 mL for ER, PR and HER2, based on ≥70% recovery in at least 7 of 8 replicates.

*Linearity and Limit of Detection (HER2 FISH)*

Linearity and Limit of Detection (LoD) were established by spiking varying amounts (1, 2, 4, 8, 16, and 32) of SKBR3 cells into 1 mL of healthy donor blood and estimating the recovery of these cells based on ERBB2/CEN17 signal detection. The study was performed independently by 2 separate users. The mean recovery was used to determine the linearity and LoD. R^2^ ≥ 0.99 indicates significant linear response of the test with a low LoD of 1 cell per 1 mL equivalent of PBMCs (S5 Fig).

**S5 Fig. Analytical Validation: Linearity (ICC and HER2-FISH).**

The Test exhibited significant linearity with R^2^ ≥0.99.


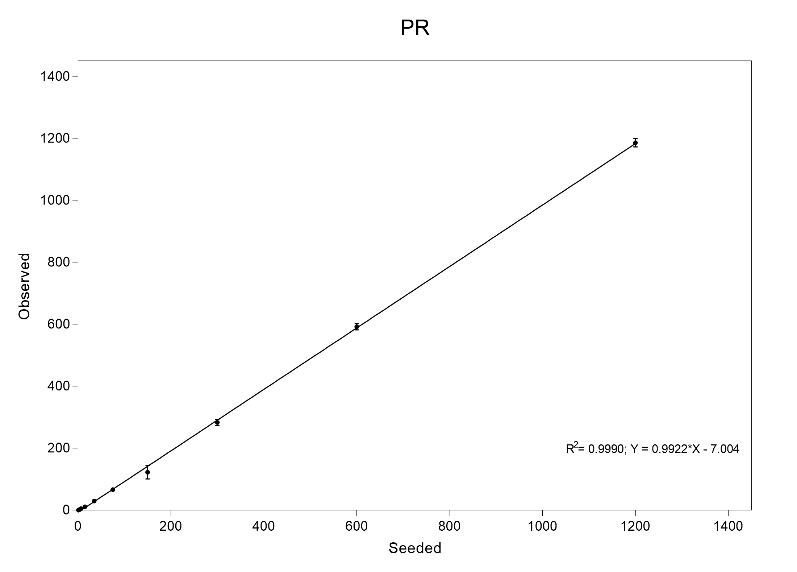

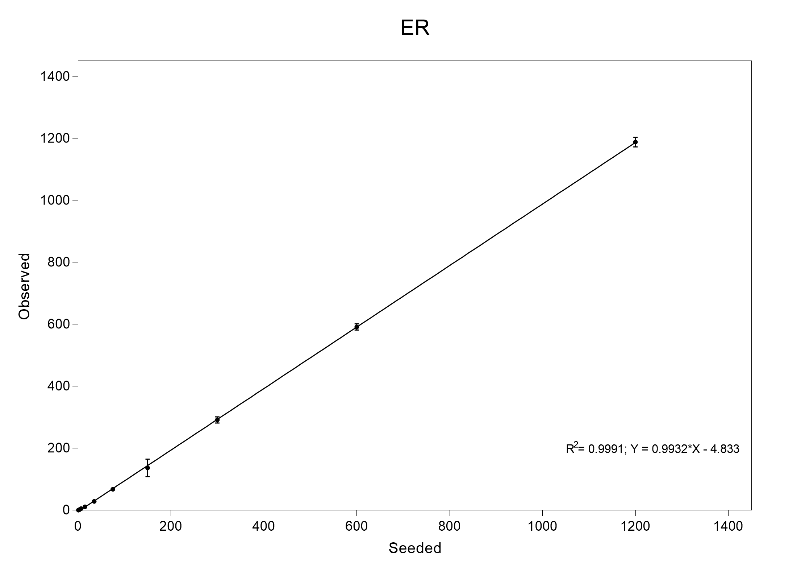

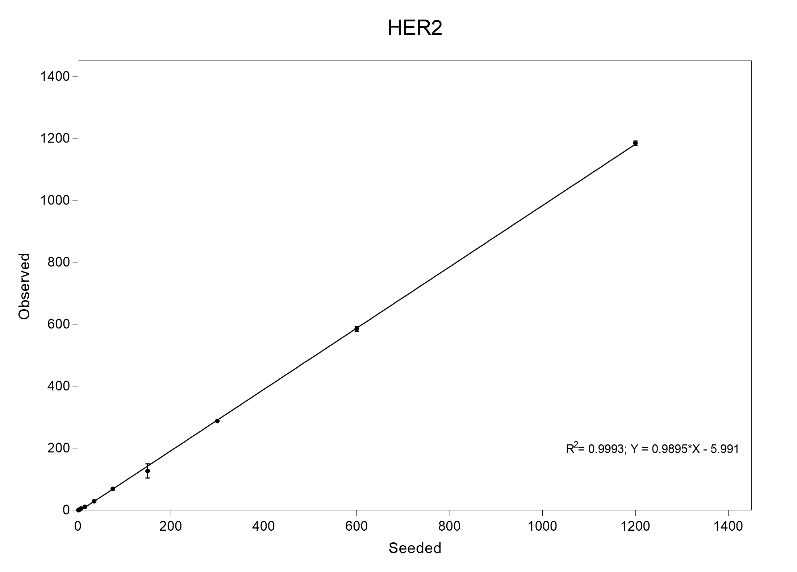

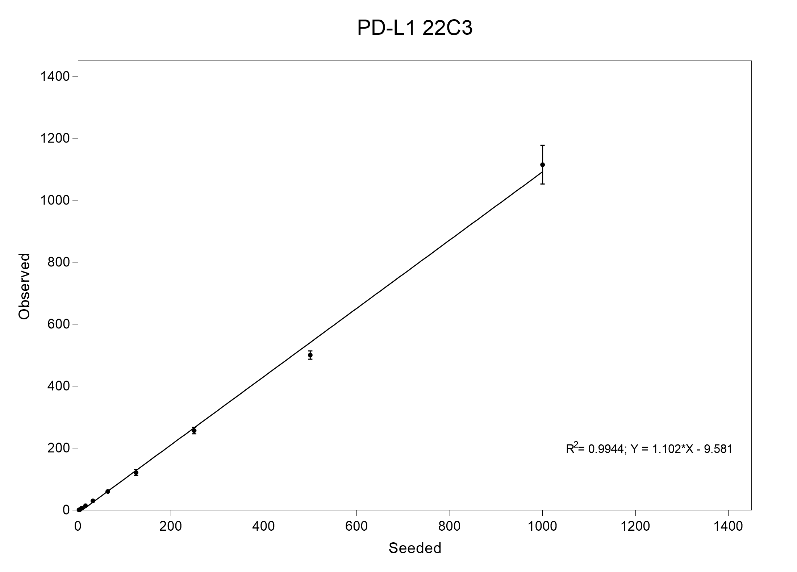

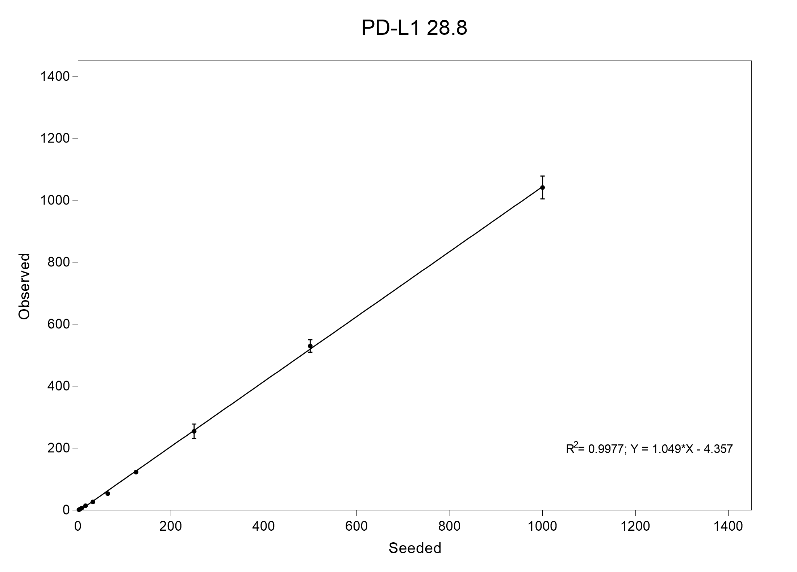

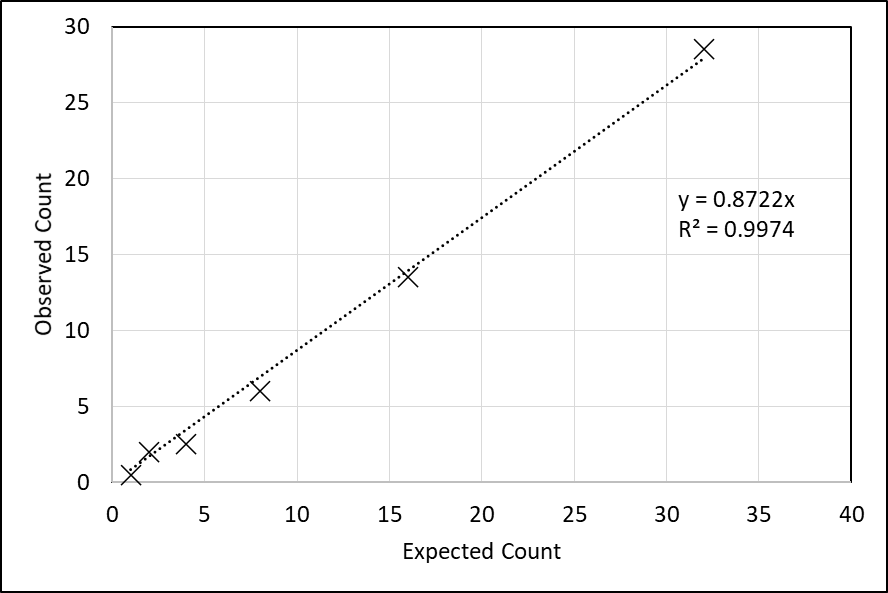


HER2 (FISH)
