# Supplementary material for: Circulating tumor cell assay to non-invasively evaluate PD-L1 and other therapeutic targets in multiple cancers
Source: PLoS One. 2022 Jun 17;17(6):e0270139. doi: 10.1371/journal.pone.0270139 (PMC9205490; doi:10.1371/journal.pone.0270139)
Supplement: S1 Table — (DOCX) [file pone.0270139.s006.docx]

**S1 Table. Antisera and Control Cells.**

| **Name** | **Catalog #** | **Vendor** |
| --- | --- | --- |
| RH Anti-CK IgG-Vio 515 | 130-112-746 | Miltenyi Biotec |
| RH Anti-CD45-IgG-APCVio 770 | 130-110-635 | Miltenyi Biotec |
| Mouse Anti-PD-L1 (22C3) | SK006 | Dako |
| Rabbit Anti-PD-L1 (28.8) | SK005 | Dako |
| Rabbit Anti-Estrogen Receptor (Ep1) | IS084 | Dako |
| Rabbit Anti-PR (SP2) | PRM302AA | Biocare Medical |
| Rabbit Anti-c-erbB-2 (HER2; polyclonal) | A0485 | Dako |
| Anti-rabbit Alexa Fluor 594 | A32740 | Invitrogen |
| Anti-mouse Alexa Fluor 594 | A32742 | Invitrogen |
| SKBR3 (Breast cancer) cells* | HTB-30™ | ATCC |
| SW982 (Synovial Sarcoma) cells* | HTB-93™ | ATCC |
| MOLT-3 (Leukemia) cells* | CRL-1552™ | ATCC |
| MCF7 (Breast cancer) cells* | HTB-22^TM^ | ATCC^#^ |
| MDA-MB-231 (Breast cancer) cells* | HTB-26™ | ATCC^#^ |
| *used as controls in the Test  ^#^ procured from ATCC via NCCS, India | | |
