# Supplementary material for: Circulating tumor cell assay to non-invasively evaluate PD-L1 and other therapeutic targets in multiple cancers
Source: PLoS One. 2022 Jun 17;17(6):e0270139. doi: 10.1371/journal.pone.0270139 (PMC9205490; doi:10.1371/journal.pone.0270139)
Supplement: S2 Table — (DOCX) [file pone.0270139.s007.docx]

**S2 Table. Details of asymptomatic samples (HER2-FISH analysis).**

|  | **Male** | **Female** | **Total** |
| --- | --- | --- | --- |
| **N =** | 10 | 10 | 20 |
| **Median Age, Range** | 31 (22 – 37) | 29 (21 – 40) | 30 (21 – 40) |
