# Supplementary material for: Circulating tumor cell assay to non-invasively evaluate PD-L1 and other therapeutic targets in multiple cancers
Source: PLoS One. 2022 Jun 17;17(6):e0270139. doi: 10.1371/journal.pone.0270139 (PMC9205490; doi:10.1371/journal.pone.0270139)
Supplement: S3 Table — (DOCX) [file pone.0270139.s008.docx]

**S3 Table. Demographics of Clinical Validation Cohort (ICC and HER2-FISH).**

|  | **PD-L1 22C3** | **PD-L1 28.8** | **ER** | **PR** | **HER2** | **HER2 (FISH)** |
| --- | --- | --- | --- | --- | --- | --- |
| **Age (years)**  Median  Range | 53  (23 – 88) | | 51  (30 - 73) | | | 54  (27 – 78) |
| **Gender**  Male  Female | 34  73 | | 6  95 | | 6  93 | 4  50 |
| **Tumor types**  Breast  Bladder  Cervix  Colon/Rectum  Esophagus  Head and Neck  Lung  Stomach  Uterus  Vulva | 48  4  6  -  6  23  13  6  -  1 | | 101  -  -  -  -  -  -  -  -  - | | 99  -  -  -  -  -  -  -  -  - | 46  -  -  3  1  -  -  2  2  - |
| **Total** | 107 | | 101 | | 99 | 54 |
