# Supplementary material for: Circulating tumor cell assay to non-invasively evaluate PD-L1 and other therapeutic targets in multiple cancers
Source: PLoS One. 2022 Jun 17;17(6):e0270139. doi: 10.1371/journal.pone.0270139 (PMC9205490; doi:10.1371/journal.pone.0270139)
Supplement: S4 Table — (DOCX) [file pone.0270139.s009.docx]

**S4 Table. Cross Validation.** The performance characteristics were determined via a 20-fold cross validation.

1. PD-L1 22C3

| **Iteration** | **Sample Type** | **Samples** | **Test Classification** | |
| --- | --- | --- | --- | --- |
|  |  |  | **PD-L1 22C3+** | **PD-L1 22C3-** |
| **Training Set** | **PD-L1 22C3+** | 20 | TP = 19 | FN = 1 |
|  | **PD-L1 22C3-** | 28 | FP = 3 | TN = 25 |
| **Test Set 1** | **PD-L1 22C3+** | 9 | TP = 9 | FN = 0 |
|  | **PD-L1 22C3-** | 12 | FP = 1 | TN = 11 |
| **Test Set 2** | **PD-L1 22C3+** | 10 | TP = 10 | FN = 0 |
|  | **PD-L1 22C3-** | 11 | FP = 0 | TN = 11 |
| **Test Set 3** | **PD-L1 22C3+** | 8 | TP = 8 | FN = 0 |
|  | **PD-L1 22C3-** | 13 | FP = 2 | TN = 11 |
| **Test Set 4** | **PD-L1 22C3+** | 8 | TP = 8 | FN = 0 |
|  | **PD-L1 22C3-** | 13 | FP = 2 | TN = 11 |
| **Test Set 5** | **PD-L1 22C3+** | 9 | TP = 9 | FN = 0 |
|  | **PD-L1 22C3-** | 12 | FP = 1 | TN = 11 |
| **Test Set 6** | **PD-L1 22C3+** | 9 | TP = 9 | FN = 0 |
|  | **PD-L1 22C3-** | 12 | FP = 1 | TN = 11 |
| **Test Set 7** | **PD-L1 22C3+** | 9 | TP = 9 | FN = 0 |
|  | **PD-L1 22C3-** | 12 | FP = 1 | TN = 11 |
| **Test Set 8** | **PD-L1 22C3+** | 10 | TP = 10 | FN = 0 |
|  | **PD-L1 22C3-** | 11 | FP = 0 | TN = 11 |
| **Test Set 9** | **PD-L1 22C3+** | 8 | TP = 8 | FN = 0 |
|  | **PD-L1 22C3-** | 13 | FP = 2 | TN = 11 |
| **Test Set 10** | **PD-L1 22C3+** | 10 | TP = 10 | FN = 0 |
|  | **PD-L1 22C3-** | 11 | FP = 0 | TN = 11 |
| **Test Set 11** | **PD-L1 22C3+** | 10 | TP = 10 | FN = 0 |
|  | **PD-L1 22C3-** | 11 | FP = 0 | TN = 11 |
| **Test Set 12** | **PD-L1 22C3+** | 10 | TP = 10 | FN = 0 |
|  | **PD-L1 22C3-** | 11 | FP = 0 | TN = 11 |
| **Test Set 13** | **PD-L1 22C3+** | 9 | TP = 9 | FN = 0 |
|  | **PD-L1 22C3-** | 12 | FP = 1 | TN = 11 |
| **Test Set 14** | **PD-L1 22C3+** | 10 | TP = 10 | FN = 0 |
|  | **PD-L1 22C3-** | 11 | FP = 0 | TN = 11 |
| **Test Set 15** | **PD-L1 22C3+** | 9 | TP = 9 | FN = 0 |
|  | **PD-L1 22C3-** | 12 | FP = 1 | TN = 11 |
| **Test Set 16** | **PD-L1 22C3+** | 9 | TP = 9 | FN = 0 |
|  | **PD-L1 22C3-** | 12 | FP = 1 | TN = 11 |
| **Test Set 17** | **PD-L1 22C3+** | 7 | TP = 7 | FN = 0 |
|  | **PD-L1 22C3-** | 14 | FP = 3 | TN = 11 |
| **Test Set 18** | **PD-L1 22C3+** | 10 | TP = 10 | FN = 0 |
|  | **PD-L1 22C3-** | 11 | FP = 0 | TN = 11 |
| **Test Set 19** | **PD-L1 22C3+** | 9 | TP = 9 | FN = 0 |
|  | **PD-L1 22C3-** | 12 | FP = 1 | TN = 11 |
| **Test Set 20** | **PD-L1 22C3+** | 9 | TP = 9 | FN = 0 |
|  | **PD-L1 22C3-** | 12 | FP = 1 | TN = 11 |

1. PD-L1 28.8

| **Iteration** | **Sample Type** | **Samples** | **Test Classification** | |
| --- | --- | --- | --- | --- |
|  |  |  | **PD-L1 28.8+** | **PD-L1 28.8-** |
| **Training Set** | **PD-L1 28.8+** | 20 | TP = 19 | FN = 1 |
|  | **PD-L1 28.8-** | 28 | FP = 3 | TN = 25 |
| **Test Set 1** | **PD-L1 28.8+** | 9 | TP = 9 | FN = 0 |
|  | **PD-L1 28.8-** | 12 | FP = 1 | TN = 11 |
| **Test Set 2** | **PD-L1 28.8+** | 10 | TP = 10 | FN = 0 |
|  | **PD-L1 28.8-** | 11 | FP = 0 | TN = 11 |
| **Test Set 3** | **PD-L1 28.8+** | 8 | TP = 8 | FN = 0 |
|  | **PD-L1 28.8-** | 13 | FP = 2 | TN = 11 |
| **Test Set 4** | **PD-L1 28.8+** | 9 | TP = 8 | FN = 1 |
|  | **PD-L1 28.8-** | 12 | FP = 2 | TN = 10 |
| **Test Set 5** | **PD-L1 28.8+** | 9 | TP = 8 | FN = 1 |
|  | **PD-L1 28.8-** | 12 | FP = 2 | TN = 10 |
| **Test Set 6** | **PD-L1 28.8+** | 9 | TP = 9 | FN = 0 |
|  | **PD-L1 28.8-** | 12 | FP = 1 | TN = 11 |
| **Test Set 7** | **PD-L1 28.8+** | 10 | TP = 10 | FN = 0 |
|  | **PD-L1 28.8-** | 11 | FP = 0 | TN = 11 |
| **Test Set 8** | **PD-L1 28.8+** | 8 | TP = 8 | FN = 0 |
|  | **PD-L1 28.8-** | 13 | FP = 2 | TN = 11 |
| **Test Set 9** | **PD-L1 28.8+** | 10 | TP = 9 | FN = 1 |
|  | **PD-L1 28.8-** | 11 | FP = 1 | TN = 10 |
| **Test Set 10** | **PD-L1 28.8+** | 10 | TP = 9 | FN = 1 |
|  | **PD-L1 28.8-** | 11 | FP = 1 | TN = 10 |
| **Test Set 11** | **PD-L1 28.8+** | 10 | TP = 9 | FN = 1 |
|  | **PD-L1 28.8-** | 11 | FP = 1 | TN = 10 |
| **Test Set 12** | **PD-L1 28.8+** | 10 | TP = 9 | FN = 1 |
|  | **PD-L1 28.8-** | 11 | FP = 1 | TN = 10 |
| **Test Set 13** | **PD-L1 28.8+** | 10 | TP = 9 | FN = 1 |
|  | **PD-L1 28.8-** | 11 | FP = 1 | TN = 10 |
| **Test Set 14** | **PD-L1 28.8+** | 10 | TP = 10 | FN = 0 |
|  | **PD-L1 28.8-** | 11 | FP = 0 | TN = 11 |
| **Test Set 15** | **PD-L1 28.8+** | 8 | TP = 8 | FN = 0 |
|  | **PD-L1 28.8-** | 13 | FP = 2 | TN =11 |
| **Test Set 16** | **PD-L1 28.8+** | 10 | TP = 9 | FN = 1 |
|  | **PD-L1 28.8-** | 11 | FP = 1 | TN = 10 |
| **Test Set 17** | **PD-L1 28.8+** | 9 | TP = 9 | FN = 0 |
|  | **PD-L1 28.8-** | 12 | FP = 1 | TN = 11 |
| **Test Set 18** | **PD-L1 28.8+** | 9 | TP = 9 | FN = 0 |
|  | **PD-L1 28.8-** | 12 | FP = 1 | TN = 11 |
| **Test Set 19** | **PD-L1 28.8+** | 7 | TP = 7 | FN = 0 |
|  | **PD-L1 28.8-** | 14 | FP = 3 | TN = 11 |
| **Test Set 20** | **PD-L1 28.8+** | 10 | TP = 9 | FN = 1 |
|  | **PD-L1 28.8-** | 11 | FP = 1 | TN = 10 |

1. ER

| **Iteration** | **Sample Type** | **Samples** | **Test Classification** | |
| --- | --- | --- | --- | --- |
|  |  |  | **ER+** | **ER-** |
| **Training Set** | **ER+** | 37 | TP = 35 | FN = 2 |
|  | **ER-** | 36 | FP = 7 | TN = 29 |
| **Test Set 1** | **ER+** | 17 | TP = 14 | FN = 3 |
|  | **ER-** | 15 | FP = 4 | TN = 11 |
| **Test Set 2** | **ER+** | 19 | TP = 16 | FN = 3 |
|  | **ER-** | 13 | FP = 2 | TN = 11 |
| **Test Set 3** | **ER+** | 14 | TP = 13 | FN = 1 |
|  | **ER-** | 18 | FP = 5 | TN = 13 |
| **Test Set 4** | **ER+** | 18 | TP = 15 | FN = 3 |
|  | **ER-** | 14 | FP = 3 | TN = 11 |
| **Test Set 5** | **ER+** | 14 | TP = 13 | FN = 1 |
|  | **ER-** | 18 | FP = 5 | TN = 13 |
| **Test Set 6** | **ER+** | 16 | TP = 15 | FN = 1 |
|  | **ER-** | 16 | FP = 3 | TN = 13 |
| **Test Set 7** | **ER+** | 17 | TP = 15 | FN = 2 |
|  | **ER-** | 15 | FP = 3 | TN = 12 |
| **Test Set 8** | **ER+** | 15 | TP = 15 | FN = 0 |
|  | **ER-** | 17 | FP = 3 | TN = 14 |
| **Test Set 9** | **ER+** | 19 | TP = 17 | FN = 2 |
|  | **ER-** | 13 | FP = 1 | TN = 12 |
| **Test Set 10** | **ER+** | 16 | TP = 15 | FN = 1 |
|  | **ER-** | 16 | FP = 3 | TN = 13 |
| **Test Set 11** | **ER+** | 16 | TP = 15 | FN = 1 |
|  | **ER-** | 16 | FP = 3 | TN = 13 |
| **Test Set 12** | **ER+** | 19 | TP = 15 | FN = 4 |
|  | **ER-** | 13 | FP = 3 | TN = 10 |
| **Test Set 13** | **ER+** | 16 | TP = 15 | FN = 1 |
|  | **ER-** | 16 | FP = 3 | TN = 13 |
| **Test Set 14** | **ER+** | 16 | TP = 16 | FN = 0 |
|  | **ER-** | 16 | FP = 2 | TN = 14 |
| **Test Set 15** | **ER+** | 18 | TP = 16 | FN = 2 |
|  | **ER-** | 14 | FP = 2 | TN = 12 |
| **Test Set 16** | **ER+** | 16 | TP = 14 | FN = 2 |
|  | **ER-** | 16 | FP = 4 | TN = 12 |
| **Test Set 17** | **ER+** | 18 | TP = 16 | FN = 2 |
|  | **ER-** | 14 | FP = 2 | TN = 12 |
| **Test Set 18** | **ER+** | 14 | TP = 13 | FN = 1 |
|  | **ER-** | 18 | FP = 5 | TN = 13 |
| **Test Set 19** | **ER+** | 15 | TP = 14 | FN = 1 |
|  | **ER-** | 17 | FP = 4 | TN = 13 |
| **Test Set 20** | **ER+** | 19 | TP = 15 | FN = 4 |
|  | **ER-** | 13 | FP = 3 | TN = 10 |

1. PR

| **Iteration** | **Sample Type** | **Samples** | **Test Classification** | |
| --- | --- | --- | --- | --- |
|  |  |  | **PR+** | **PR-** |
| **Training Set** | **PR+** | 32 | TP = 29 | FN = 3 |
|  | **PR-** | 42 | FP = 6 | TN = 36 |
| **Test Set 1** | **PR+** | 10 | TP = 9 | FN = 1 |
|  | **PR-** | 21 | FP = 6 | TN = 15 |
| **Test Set 2** | **PR+** | 15 | TP = 14 | FN = 1 |
|  | **PR-** | 16 | FP = 1 | TN = 15 |
| **Test Set 3** | **PR+** | 12 | TP = 12 | FN = 0 |
|  | **PR-** | 19 | FP = 3 | TN = 16 |
| **Test Set 4** | **PR+** | 14 | TP = 13 | FN = 1 |
|  | **PR-** | 17 | FP = 2 | TN = 15 |
| **Test Set 5** | **PR+** | 15 | TP = 13 | FN = 2 |
|  | **PR-** | 16 | FP = 2 | TN = 14 |
| **Test Set 6** | **PR+** | 14 | TP = 12 | FN = 2 |
|  | **PR-** | 17 | FP = 3 | TN = 14 |
| **Test Set 7** | **PR+** | 14 | TP = 13 | FN = 1 |
|  | **PR-** | 17 | FP = 2 | TN = 15 |
| **Test Set 8** | **PR+** | 14 | TP = 14 | FN = 0 |
|  | **PR-** | 17 | FP = 1 | TN = 16 |
| **Test Set 9** | **PR+** | 12 | TP = 11 | FN = 1 |
|  | **PR-** | 19 | FP = 4 | TN = 15 |
| **Test Set 10** | **PR+** | 10 | TP = 10 | FN = 0 |
|  | **PR-** | 21 | FP = 5 | TN = 16 |
| **Test Set 11** | **PR+** | 11 | TP = 10 | FN = 1 |
|  | **PR-** | 20 | FP = 5 | TN = 15 |
| **Test Set 12** | **PR+** | 15 | TP = 13 | FN = 2 |
|  | **PR-** | 16 | FP = 2 | TN = 14 |
| **Test Set 13** | **PR+** | 13 | TP = 12 | FN = 1 |
|  | **PR-** | 18 | FP = 3 | TN = 15 |
| **Test Set 14** | **PR+** | 15 | TP = 14 | FN = 1 |
|  | **PR-** | 16 | FP = 1 | TN = 15 |
| **Test Set 15** | **PR+** | 11 | TP = 10 | FN = 1 |
|  | **PR-** | 20 | FP = 5 | TN = 15 |
| **Test Set 16** | **PR+** | 12 | TP = 11 | FN = 1 |
|  | **PR-** | 19 | FP = 4 | TN = 15 |
| **Test Set 17** | **PR+** | 12 | TP = 11 | FN =1 |
|  | **PR-** | 19 | FP = 4 | TN = 15 |
| **Test Set 18** | **PR+** | 12 | TP = 11 | FN = 1 |
|  | **PR-** | 19 | FP = 4 | TN = 15 |
| **Test Set 19** | **PR+** | 12 | TP = 12 | FN = 0 |
|  | **PR-** | 19 | FP = 3 | TN = 16 |
| **Test Set 20** | **PR+** | 13 | TP = 12 | FN = 1 |
|  | **PR-** | 18 | FP = 3 | TN = 15 |

1. HER2 (ICC)

| **Iteration** | **Sample Type** | **Samples** | **Test Classification** | |
| --- | --- | --- | --- | --- |
|  |  |  | **HER2+** | **HER2-** |
| **Training Set** | **HER2+** | 16 | TP = 12 | FN = 4 |
|  | **HER2-** | 45 | FP = 6 | TN = 39 |
| **Test Set 1** | **HER2+** | 8 | TP = 5 | FN = 3 |
|  | **HER2-** | 18 | FP = 3 | TN = 15 |
| **Test Set 2** | **HER2+** | 6 | TP = 3 | FN = 3 |
|  | **HER2-** | 20 | FP = 5 | TN = 15 |
| **Test Set 3** | **HER2+** | 7 | TP = 5 | FN = 2 |
|  | **HER2-** | 19 | FP = 3 | TN = 16 |
| **Test Set 4** | **HER2+** | 7 | TP = 6 | FN = 1 |
|  | **HER2-** | 19 | FP = 2 | TN = 17 |
| **Test Set 5** | **HER2+** | 8 | TP = 6 | FN = 2 |
|  | **HER2-** | 18 | FP = 2 | TN = 16 |
| **Test Set 6** | **HER2+** | 10 | TP = 6 | FN = 4 |
|  | **HER2-** | 16 | FP = 2 | TN = 14 |
| **Test Set 7** | **HER2+** | 7 | TP = 4 | FN = 3 |
|  | **HER2-** | 19 | FP = 4 | TN = 15 |
| **Test Set 8** | **HER2+** | 7 | TP = 4 | FN = 3 |
|  | **HER2-** | 19 | FP = 4 | TN = 15 |
| **Test Set 9** | **HER2+** | 7 | TP = 5 | FN = 2 |
|  | **HER2-** | 19 | FP = 3 | TN = 16 |
| **Test Set 10** | **HER2+** | 7 | TP = 4 | FN = 3 |
|  | **HER2-** | 19 | FP = 4 | TN = 15 |
| **Test Set 11** | **HER2+** | 8 | TP = 4 | FN = 4 |
|  | **HER2-** | 18 | FP = 4 | TN = 14 |
| **Test Set 12** | **HER2+** | 3 | TP = 3 | FN = 0 |
|  | **HER2-** | 23 | FP = 5 | TN = 18 |
| **Test Set 13** | **HER2+** | 10 | TP = 5 | FN = 5 |
|  | **HER2-** | 16 | FP = 3 | TN = 13 |
| **Test Set 14** | **HER2+** | 6 | TP = 6 | FN = 0 |
|  | **HER2-** | 20 | FP = 2 | TN = 18 |
| **Test Set 15** | **HER2+** | 10 | TP = 8 | FN = 2 |
|  | **HER2-** | 16 | FP = 0 | TN = 16 |
| **Test Set 16** | **HER2+** | 6 | TP = 5 | FN = 1 |
|  | **HER2-** | 20 | FP = 3 | TN = 17 |
| **Test Set 17** | **HER2+** | 8 | TP = 5 | FN = 3 |
|  | **HER2-** | 18 | FP = 3 | TN = 15 |
| **Test Set 18** | **HER2+** | 5 | TP = 4 | FN = 1 |
|  | **HER2-** | 21 | FP = 4 | TN = 17 |
| **Test Set 19** | **HER2+** | 6 | TP = 4 | FN = 2 |
|  | **HER2-** | 20 | FP = 4 | TN = 16 |
| **Test Set 20** | **HER2+** | 6 | TP = 5 | FN = 1 |
|  | **HER2-** | 20 | FP = 3 | TN = 17 |

1. HER2 (FISH)

| **Iteration** | **Sample Type** | **Samples** | **Test Classification (HER2 FISH)** | |
| --- | --- | --- | --- | --- |
|  |  |  | **HER2+** | **HER2-** |
| **Training Set** | **HER2+** | 9 | TP = 8 | FN = 1 |
|  | **HER2-** | 29 | FP = 1 | TN = 28 |
| **Test Set 1** | **HER2+** | 5 | TP = 4 | FN = 1 |
|  | **HER2-** | 11 | FP = 0 | TN = 11 |
| **Test Set 2** | **HER2+** | 3 | TP = 3 | FN = 0 |
|  | **HER2-** | 13 | FP = 1 | TN = 12 |
| **Test Set 3** | **HER2+** | 4 | TP = 4 | FN = 0 |
|  | **HER2-** | 12 | FP = 0 | TN = 12 |
| **Test Set 4** | **HER2+** | 5 | TP = 4 | FN = 1 |
|  | **HER2-** | 11 | FP = 0 | TN = 11 |
| **Test Set 5** | **HER2+** | 4 | TP = 4 | FN = 0 |
|  | **HER2-** | 12 | FP = 0 | TN = 12 |
| **Test Set 6** | **HER2+** | 4 | TP = 4 | FN = 0 |
|  | **HER2-** | 12 | FP = 0 | TN = 12 |
| **Test Set 7** | **HER2+** | 4 | TP = 3 | FN = 1 |
|  | **HER2-** | 12 | FP = 1 | TN = 11 |
| **Test Set 8** | **HER2+** | 4 | TP = 4 | FN = 0 |
|  | **HER2-** | 12 | FP = 0 | TN = 12 |
| **Test Set 9** | **HER2+** | 6 | TP = 4 | FN = 2 |
|  | **HER2-** | 10 | FP = 0 | TN = 10 |
| **Test Set 10** | **HER2+** | 5 | TP = 4 | FN = 1 |
|  | **HER2-** | 11 | FP = 0 | TN = 11 |
| **Test Set 11** | **HER2+** | 4 | TP = 4 | FN = 0 |
|  | **HER2-** | 12 | FP = 0 | TN = 12 |
| **Test Set 12** | **HER2+** | 5 | TP = 4 | FN = 1 |
|  | **HER2-** | 11 | FP = 0 | TN = 11 |
| **Test Set 13** | **HER2+** | 5 | TP = 4 | FN = 1 |
|  | **HER2-** | 11 | FP = 0 | TN = 11 |
| **Test Set 14** | **HER2+** | 4 | TP = 4 | FN = 0 |
|  | **HER2-** | 12 | FP = 0 | TN = 12 |
| **Test Set 15** | **HER2+** | 4 | TP = 4 | FN = 0 |
|  | **HER2-** | 12 | FP = 0 | TN = 12 |
| **Test Set 16** | **HER2+** | 4 | TP = 3 | FN = 1 |
|  | **HER2-** | 12 | FP = 1 | TN = 11 |
| **Test Set 17** | **HER2+** | 5 | TP = 4 | FN = 1 |
|  | **HER2-** | 11 | FP = 0 | TN = 11 |
| **Test Set 18** | **HER2+** | 5 | TP = 4 | FN = 1 |
|  | **HER2-** | 11 | FP = 0 | TN = 11 |
| **Test Set 19** | **HER2+** | 5 | TP = 3 | FN = 2 |
|  | **HER2-** | 11 | FP = 1 | TN = 10 |
| **Test Set 20** | **HER2+** | 4 | TP = 4 | FN = 0 |
|  | **HER2-** | 12 | FP = 0 | TN = 12 |
