# Supplementary material for: Circulating tumor cell assay to non-invasively evaluate PD-L1 and other therapeutic targets in multiple cancers
Source: PLoS One. 2022 Jun 17;17(6):e0270139. doi: 10.1371/journal.pone.0270139 (PMC9205490; doi:10.1371/journal.pone.0270139)
Supplement: S5 Table — (DOCX) [file pone.0270139.s010.docx]

**S5 Table. Clinical validation (HER2 FISH Cohort 2) - Three-way concordance study.**

N = 33 samples, Female, Breast Cancer, Median age = 55 years (40 - 78)

| **IHC** | **Tissue FISH** | | **CTAC FISH** | |
| --- | --- | --- | --- | --- |
|  | **Positive** | **Negative** | **Positive** | **Negative** |
| **Positive = 10** | 8 (80%) | 2 | 9 (90%) | 1 |
| **Negative = 25** | 0 | 25 (100%) | 1 | 24 (96%) |
| **Equivocal = 9** | 2 | 7 (77.8%) | 2 | 7 (77.8%) |

Similar definitions as in analytical validation applied to clinical validation studies where known marker positivity or negativity in tissue was used as the standard:

Tissue positive, CTC positive = TP

Tissue positive, CTC negative = FN

Tissue negative, CTC positive = FP

Tissue negative, CTC negative = TN
