# Supplementary material for: Circulating tumor cell assay to non-invasively evaluate PD-L1 and other therapeutic targets in multiple cancers
Source: PLoS One. 2022 Jun 17;17(6):e0270139. doi: 10.1371/journal.pone.0270139 (PMC9205490; doi:10.1371/journal.pone.0270139)
Supplement: S7 Table — (DOCX) [file pone.0270139.s012.docx]

**Method Development and Optimization**

*Antibody Dilutions*

MDA-MB-231 was used as reference cell line for PD-L1 22C3 and PD-L1 28.8 (1,2), MCF7 for ER, PR (3–5) ; and SKBR3 for HER2 (6,7), PanCK. Respective reference cell lines were used for optimizing dilutions of antibodies (Ab) against PD-L1 22C3, PD-L1 28.8, ER, PR, HER2 and PanCK. Three different dilutions were evaluated for each Ab with 6 replicates per dilution. FI of each marker was compared at each dilution to determine the lowest concentration with higher FI. The evaluated dilutions, optimal dilutions, list of markers, fluorophores and detection (excitation and emission spectra) are provided in S6 and S7 Tables.

**S7 Table. Optimization of antibody dilution.** All Ab were evaluated at 3 different dilutions. The optimal dilutions are as indicated in bold text.

| **Antibody** | **Dilution 1** | **Dilution 2** | **Dilution 3** |
| --- | --- | --- | --- |
| **Anti-PD-L1 22C3** | Undiluted | 1:2 | **1:4** |
| **Anti-PD-L1 28.8** | Undiluted | 1:2 | **1:4** |
| **Anti-ER** | Undiluted | 1:2 | **1:4** |
| **Anti-PR** | Undiluted | 1:2 | **1:4** |
| **Anti-HER2** | 1:700 | 1:1400 | **1:2800** |
| **Anti-PanCK** | 1:400 | **1:500** | 1:600 |
| **Anti-rabbit AF 594** | 1:250 | **1:500** | 1:1000 |
| **Anti-mouse AF 594** | 1:250 | **1:500** | 1:1000 |
| **Anti-CD45** | 1:250 | **1:500** | 1:1000 |

**References**

1. Ghebeh H, Mohammed S, Al-Omair A, Qattan A, Lehe C, Al-Qudaihi G, et al. The B7-H1 (PD-L1) T lymphocyte-inhibitory molecule is expressed in breast cancer patients with infiltrating ductal carcinoma: correlation with important high-risk prognostic factors. Neoplasia. 2006 Mar;8(3):190–8.

2. Mittendorf EA, Philips A V, Meric-Bernstam F, Qiao N, Wu Y, Harrington S, et al. PD-L1 expression in triple-negative breast cancer. Cancer Immunol Res. 2014 Apr;2(4):361–70.

3. Comşa Ş, Cîmpean AM, Raica M. The Story of MCF-7 Breast Cancer Cell Line: 40 years of Experience in Research. Anticancer Res. 2015 Jun;35(6):3147–54.

4. Ford CHJ, Al-Bader M, Al-Ayadhi B, Francis I. Reassessment of estrogen receptor expression in human breast cancer cell lines. Anticancer Res. 2011 Feb;31(2):521–7.

5. Clare SE, Gupta A, Choi M, Ranjan M, Lee O, Wang J, et al. Progesterone receptor blockade in human breast cancer cells decreases cell cycle progression through G2/M by repressing G2/M genes. BMC Cancer. 2016 May;16:326.

6. Holliday DL, Speirs V. Choosing the right cell line for breast cancer research. Breast Cancer Res. 2011 Aug;13(4):215.

7. Ren W, Liu Y, Wan S, Fei C, Wang W, Chen Y, et al. BMP9 inhibits proliferation and metastasis of HER2-positive SK-BR-3 breast cancer cells through ERK1/2 and PI3K/AKT pathways. PLoS One. 2014;9(5):e96816.
