# Supplementary material for: Circulating tumor cell assay to non-invasively evaluate PD-L1 and other therapeutic targets in multiple cancers
Source: PLoS One. 2022 Jun 17;17(6):e0270139. doi: 10.1371/journal.pone.0270139 (PMC9205490; doi:10.1371/journal.pone.0270139)
Supplement: S8 Table — (DOCX) [file pone.0270139.s013.docx]

**Analytical Validation - ICC**

*Linearity*

Recoveries of spiked cells were generally higher at higher spike densities (8 cells / 5 ml for PD-L1 22C3 and 28.8; 7 cells/ 5 ml for ER, PR, HER2) (S5 Fig, S8 Table). R^2^ ≥0.99 in all markers indicated a significant linear response, especially in the range of 7 – 1000 cells / 5 mL for PD-L1 and 7 – 1200 cells / 5 mL for ER, PR and HER2.

**S8 Table. Analytical Validation: Linearity.** The Test exhibited significant linearity with R^2^ ≥0.99. The Tabulated values below show the recovery and range of recovery.

| **Spiked  Cells** | **Mean, % and Range of Detected Cell Counts** | |
| --- | --- | --- |
|  | **PD-L1 22C3** | **PD-L1 28.8** |
| 0 | 0 | 0 |
| 2 | 1 (50%)  (0-2) | 1 (50%)  (1-2) |
| 4 | 2 (50%)  (0-4) | 4 (100%)  (0-7) |
| 8 | 7 (87.5%)  (5-9) | 7 (87.5%)  (5-10) |
| 16 | 14 (87.5%)  (11-20) | 14 (87.5%)  (12-20) |
| 32 | 30 (93.8%)  (21-35) | 27 (84.4%)  (25-29) |
| 64 | 60 (93.8%)  (55-65) | 53 (82.8%)  (52-55) |
| 125 | 121 (96.8%)  (104-135) | 123 (98.4%)  (120-127) |
| 250 | 257 (102.8%)  (240-275) | 255 (102%)  (236-312) |
| 500 | 501 (100.2%)  (485-531) | 530 (106%)  (510-570) |
| 1000 | 1115 (111.5%)  (1025-1189) | 1041 (104.1%)  (1006-1125) |

| **Spiked  Cells** | **Mean, % and Range of Detected Cell Counts** | | |
| --- | --- | --- | --- |
|  | **ER** | **PR** | **HER2** |
| 0 | 0 | 0 | 0 |
| 1 | 0 (0%)  (0-0) | 0 (0%)  (0-0) | 0 (0%)  (0-0) |
| 3 | 2 (66.7%)  (1-4) | 2 (66.7%)  (1-3) | 2 (66.7%)  (0-3) |
| 7 | 5 (71.4%)  (2-7) | 5 (71.4%)  (2-6) | 6 (85.7%)  (5-7) |
| 15 | 11 (73.3%)  (9-14) | 11 (73.3%)  (8-15) | 11 (73.3%)  (8-14) |
| 35 | 28 (80%)  (25-32) | 30 (85.7%)  (27-32) | 29 (82.9%)  (26-32) |
| 75 | 67 (89.3%)  (65-70) | 66 (88%)  (59-72) | 69 (92%)  (61-72) |
| 150 | 136 (90.7%)  (82-173) | 123 (82%)  (75-144) | 127 (84.7%)  (75-154) |
| 300 | 292 (97.3%)  (270-302) | 283 (94.3%)  (269-298) | 288 (96%)  (280-295) |
| 600 | 592 (98.7%)  (578-605) | 592 (98.7%)  (578-610) | 586 (97.7%)  (570-594) |
| 1200 | 1188 (99%)  (1158-1205) | 1186 (98.8%)  (1165-1203) | 1185 (98.8%)  (1172-1198) |
