# Supplementary material for: Circulating tumor cell assay to non-invasively evaluate PD-L1 and other therapeutic targets in multiple cancers
Source: PLoS One. 2022 Jun 17;17(6):e0270139. doi: 10.1371/journal.pone.0270139 (PMC9205490; doi:10.1371/journal.pone.0270139)
Supplement: S9 Table — (DOCX) [file pone.0270139.s014.docx]

**Analytical Validation - ICC**

*Stability and Recovery*

Recoveries of various marker positive cells in the spiked samples are provided in S9 Table. Higher recoveries (>80%) were observed up to 48h for all markers, (except HER2 which showed >80% recovery only up to 24h), which appeared to be the limit for analyte stability. Similarly, in clinical samples, the recovery of marker positive cells (S10 Table) was >80% up to 48h which appeared to be the limit for analyte stability. The findings of the stability and recovery study indicated that the samples could be stored at 2°C-8°C for up to 48h with <20% loss of cells (except for PD-L1 28.8, which could be stored for up to 24h).

**S9 Table. Analytical Validation: Analyte Stability and Recovery (Spiked Cells)**. Reference cells were spiked into healthy donor blood samples and the recovery of spiked cells was evaluated for up to 120 hours.

| **Time (h)** | **Spiked**  **Cells** | **Mean Recovery, % Recovery and Recovery Range (%)** | | | | |
| --- | --- | --- | --- | --- | --- | --- |
|  |  | **PD-L1 22C3+** | **PD-L1 28.8+** | **ER+** | **PR+** | **HER2+** |
| 0 | 15 | 14 (93.3%)  (13--15) | 14.3 (95.6%)  (14--15) | 14.3 (95.6%)  (14--15) | 14.7 (97.8%)  (14--15) | 14 (93.3%)  (13--15) |
| 24 | 15 | 13.7 (91.1%)  (13--14) | 13.3 (88.9%)  (13--14) | 13.3 (88.9%)  (13--14) | 13.7 (91.1%)  (13--14) | 13 (86.7%)  (12--14) |
| 48 | 15 | 13 (86.7%)  (12--14) | 12.3 (82.2%)  (12--13) | 12.7 (84.4%)  (12--14) | 12.3 (82.2%)  (12--13) | 11.7 (77.8%)  (11--12) |
| 72 | 15 | 12.3 (82.2%)  (11--13) | 11 (73.3%)  (10--12) | 12.7 (84.4%)  (12--13) | 10.7 (71.1%)  (10--12) | 11 (73.3%)  (10--12) |
| 96 | 15 | 11.3 (75.6%)  (10--12) | 9.7 (64.4%)  (9--10) | 10.7 (71.1%)  (10--11) | 10 (66.7%)  (9--11) | 10 (66.7%)  (9--11) |
| 120 | 15 | 8.7 (57.8%)  (8--9) | 8.3 (55.6%)  (8--9) | 9.7 (64.4%)  (9--10) | 8.7 (57.8%)  (8--9) | 9 (60%)  (8--10) |
