# Supplementary material for: Circulating tumor cell assay to non-invasively evaluate PD-L1 and other therapeutic targets in multiple cancers
Source: PLoS One. 2022 Jun 17;17(6):e0270139. doi: 10.1371/journal.pone.0270139 (PMC9205490; doi:10.1371/journal.pone.0270139)
Supplement: S10 Table — (DOCX) [file pone.0270139.s015.docx]

**Analytical Validation - ICC**

*Stability and Recovery*

Recoveries of various marker positive cells in the spiked samples are provided in S9 Table. Higher recoveries (>80%) were observed up to 48h for all markers, (except HER2 which showed >80% recovery only up to 24h), which appeared to be the limit for analyte stability. Similarly, in clinical samples, the recovery of marker positive cells (S10 Table) was >80% up to 48h which appeared to be the limit for analyte stability. The findings of the stability and recovery study indicated that the samples could be stored at 2°C-8°C for up to 48h with <20% loss of cells (except for PD-L1 28.8, which could be stored for up to 24h).

**S10 Table. Analytical Validation: Analyte Stability and Recovery (CTCs)**.

Blood samples from known CTC positive cases were evaluated for recovery of CTCs for up to 120 hours.

| **Recovery of marker positive cells** | | | | | |
| --- | --- | --- | --- | --- | --- |
| **Time (h)** | **PD-L1 22C3+** | **PD-L1 28.8+** | **ER+** | **PR+** | **HER2+** |
| **0** | 8 | 7 | 6 | 5 | 6 |
| **24** | 8 (100%) | 6 (85.7%) | 6 (100%) | 4 (80%) | 5 (83.3%) |
| **48** | 7 (87.5%) | 5 (71.4%) | 5 (83.3%) | 4 (80%) | 5 (83.3%) |
| **72** | 6 (75%) | 5 (71.4%) | 4 (66.7%) | 3 (60%) | 4 (66.7%) |
| **96** | 5 (62.5%) | 4 (57.1%) | 2 (33.3%) | 2 (40%) | 3 (50%) |
| **120** | 3 (37.5%) | 3 (42.9%) | 2 (33.3%) | 2 (40%) | 2 (33.3%) |
