# Supplementary material for: Circulating tumor cell assay to non-invasively evaluate PD-L1 and other therapeutic targets in multiple cancers
Source: PLoS One. 2022 Jun 17;17(6):e0270139. doi: 10.1371/journal.pone.0270139 (PMC9205490; doi:10.1371/journal.pone.0270139)
Supplement: S11 Table — (DOCX) [file pone.0270139.s016.docx]

**Analytical Validation - ICC**

*Sensitivity, Specificity and Accuracy*

Analytical sensitivity was defined as the ability of the test to yield a positive result in the presence of the analyte (reference cells). i.e., the proportion of spiked samples where marker positive cells were detected, or the True Positive Rate.

Sensitivity = (TP / (TP + FN)) × 100 (%)

Analytical specificity was defined as the ability of the test to yield a negative result in absence of the analyte (reference cells). i.e., the proportion of unspiked samples where marker positive cells were undetectable, or the True Negative Rate.

Specificity = (TN / (TN + FP)) × 100 (%)

Analytical accuracy was defined as combined ability of the test to yield true positive and true negative findings.

Accuracy = ((TP + TN) / (TP + TN + FP + FN)) × 100 (%)

Where TP = True Positive, TN = True Negative, FP = False Positive and FN = False Negative.

Based on recovery of spiked marker positive cells, the analytical sensitivity for PD-L1 22C3 and PD-L1 28.8 was 87.5% and 90% respectively, while that of ER, PR and HER2 was determined to be 87.5%, 95% and 92.5% respectively. Since cells were undetectable in any of the 25 un-spiked samples, the analytical specificity for all the markers was deemed to be 100%. Accuracy, determined as the combined proportion of true positives and true negatives, was 92.31% for PD-L1 22C3, 93.85% for PD-L1 28.8, 92.31% for ER, 96.92% for PR and 95.38% for HER2 (Table 1 and S11 Table).

**S11 Table. Analytical Validation: Sensitivity, Specificity, Accuracy.** Reference cells were spiked into healthy donor blood samples at various seed densities and their recoveries evaluated to determine Sensitivity. Unspiked healthy donor blood samples were evaluated for false positives to determine Specificity. Accuracy was determined from Sensitivity and Specificity.

| **Spiked cells** | **Mean (Range) of Cells Detected** | **Negatives** | **Sensitivity** | **Specificity** |
| --- | --- | --- | --- | --- |
| **PD-L1 22C3+** | | | | |
| **0** | - | - | - | **100%** |
| **6** | 4 (4-5) | 5 | 37.5% | - |
| **12** | 11 (9-12) | - | 100.0% | - |
| **25** | 20 (17-22) | - | 100.0% | - |
| **50** | 41 (37-44) | - | 100.0% | - |
| **100** | 82 (80-85) | - | 100.0% | - |
| **Overall (Spiked)** | - | 5 | **87.5%** | - |
| **PD-L1 28.8+** | | | | |
| **0** | - | - | - | **100%** |
| **6** | 5 (4-5) | 4 | 50.0% | - |
| **12** | 11 (9-12) | - | 100.0% | - |
| **25** | 19 (17-21) | - | 100.0% | - |
| **50** | 40 (38-42) | - | 100.0% | - |
| **100** | 81 (78-84) | - | 100.0% | - |
| **Overall (Spiked)** | - | 4 | **90.0%** | - |
| **ER+** | | | | |
| **0** | - | - | - | **100%** |
| **6** | 5 (4-6) | 5 | 37.5% | - |
| **12** | 11 (10-12) | - | 100.0% | - |
| **25** | 21 (20-23) | - | 100.0% | - |
| **50** | 41 (39-45) | - | 100.0% | - |
| **100** | 87 (81-92) | - | 100.0% | - |
| **Overall (Spiked)** | - | 5 | **87.5%** | - |
| **PR+** | | | | |
| **0** | - | - | - | **100%** |
| **6** | 5 (4-6) | 2 | 75.0% | - |
| **12** | 10 (9-11) | - | 100.0% | - |
| **25** | 21 (19-25) | - | 100.0% | - |
| **50** | 42 (40-43) | - | 100.0% | - |
| **100** | 84 (81-85) | - | 100.0% | - |
| **Overall (Spiked)** | - | 2 | **95.0%** | - |
| **HER2+** | | | | |
| **0** | - | - | - | **100%** |
| **6** | 5 (3-5) | 3 | 62.5% | - |
| **12** | 10 (8-11) | - | 100.0% | - |
| **25** | 21 (19-22) | - | 100.0% | - |
| **50** | 42 (40-43) | - | 100.0% | - |
| **100** | 84 (81-86) | - | 100.0% | - |
| **Overall (Spiked)** | - | 3 | **92.5%** | - |
