# Supplementary material for: Circulating tumor cell assay to non-invasively evaluate PD-L1 and other therapeutic targets in multiple cancers
Source: PLoS One. 2022 Jun 17;17(6):e0270139. doi: 10.1371/journal.pone.0270139 (PMC9205490; doi:10.1371/journal.pone.0270139)
Supplement: S12 Table — (DOCX) [file pone.0270139.s017.docx]

**Analytical Validation - ICC**

*Precision*

Precision of the test was determined across 2 operators in samples with high and low spike densities of (marker positive) reference cells. S12 Table provides the %CV for intra-run, inter-run and inter-operator precision for low and high spike as well as the cumulative. The overall %CV among the low spiked samples was in the range of 3-5%, which resulted in 95-97% reproducibility while %CV among the high spiked samples was in the range of 1.5 to 2% which resulted in 98 to 99% reproducibility with all types of variation. The test showed CV <3% for all markers, PD-L1 22C3, PD-L1 28.8, ER, PR and HER2, indicating high intra-run, inter-run and inter-operator precision.

Precision of the test was defined as the closeness of multiple observations when performed under the same conditions, by same or different operators, on the same or different days, using the same or different instruments. Precision was established by showing a low coefficient of variation (CV, %) between multiple replicates of the same sample evaluated by the same / different operator on the same or different days, using the same or different instruments. For precision, mean and standard deviation (SD) of multiple measurements was obtained by standard formulae in MS Excel.

Mean = (Sum of Observation) / {Total numbers of Observations}

SD= √ *Σ* ((X-x)2/n-1), where X is the value in data distribution, x is the sample mean, n = number of observations.

The mean and SD were used to derive CV = (SD / Mean) × 100 (%)

%CV was also estimated for interference and robustness.

**S12 Table. Analytical Validation: Precision.** Recovery of low spiked reference cells in healthy donor blood samples across multiple replicates by 2 users over multiple days were used to determine the %CV.

|  |  | **Low Spike (15 cells)** | | | **High Spike (150 cells)** | | | **Overall CV%** |
| --- | --- | --- | --- | --- | --- | --- | --- | --- |
|  |  | **Mean** | **SD** | **CV%** | **Mean** | **SD** | **CV%** |  |
| **PD-L1 22C3** | **Intra-Run** | | | | | | | |
|  | User 1 | 14.71 | 0.53 | **3.59%** | 149.31 | 2.3 | **1.54%** | **2.57%** |
|  | User 2 | 14.69 | 0.48 | **3.29%** | 149.06 | 2.48 | **1.66%** | **2.48%** |
|  | Cumulative | 14.7 | 0.5 | **3.44%** | 149.18 | 2.39 | **1.60%** | **2.52%** |
|  | **Inter-Run** | | | | | | | |
|  | User 1 | 14.73 | 0.52 | **3.52%** | 149.43 | 2.37 | **1.59%** | **2.56%** |
|  | User 2 | 14.7 | 0.48 | **3.28%** | 149.06 | 2.48 | **1.66%** | **2.47%** |
|  | Cumulative | 14.71 | 0.5 | **3.40%** | 149.24 | 2.42 | **1.63%** | **2.52%** |
|  | **Inter-User** | | | | | | | |
|  | Inter-User | 14.7 | 0.51 | **3.44%** | 149.19 | 2.39 | **1.60%** | **2.52%** |
|  | **OVERALL** | - | - | **3.42%** | - | - | **1.61%** | **2.52%** |
|  |  | | | | | | | |
| **PD-L1 28.8** | **Intra-Run** | | | | | | | |
|  | User 1 | 14.8 | 0.51 | **3.47%** | 146.81 | 2.07 | **1.41%** | **2.44%** |
|  | User 2 | 14.64 | 0.48 | **3.28%** | 147 | 2.4 | **1.63%** | **2.46%** |
|  | Cumulative | 14.72 | 0.49 | **3.38%** | 146.9 | 2.23 | **1.52%** | **2.45%** |
|  | **Inter-Run** | | | | | | | |
|  | User 1 | 14.78 | 0.51 | **3.43%** | 146.93 | 2.21 | **1.50%** | **2.47%** |
|  | User 2 | 14.64 | 0.48 | **3.28%** | 147 | 2.4 | **1.63%** | **2.46%** |
|  | Cumulative | 14.71 | 0.49 | **3.36%** | 146.96 | 2.3 | **1.57%** | **2.47%** |
|  | **Inter-User** | | | | | | | |
|  | Inter-User | 14.72 | 0.5 | **3.37%** | 146.91 | 2.24 | **1.52%** | **2.45%** |
|  | **OVERALL** | - | - | **3.37%** | - | - | **1.54%** | **2.46%** |
|  |  | | | | | | | |
| **ER** | **Intra-Run** | | | | | | | |
|  | User 1 | 14.56 | 0.62 | **4.26%** | 149.15 | 2.17 | **1.46%** | **2.86%** |
|  | User 2 | 14.4 | 0.63 | **4.40%** | 149.25 | 2.27 | **1.52%** | **2.96%** |
|  | Cumulative | 14.48 | 0.62 | **4.33%** | 149.2 | 2.22 | **1.49%** | **2.91%** |
|  | **Inter-Run** | | | | | | | |
|  | User 1 | 14.58 | 0.62 | **4.23%** | 149.15 | 2.17 | **1.46%** | **2.85%** |
|  | User 2 | 14.4 | 0.63 | **4.40%** | 149.23 | 2.28 | **1.53%** | **2.97%** |
|  | Cumulative | 14.49 | 0.62 | **3.85%** | 149.19 | 2.22 | **1.50%** | **2.68%** |
|  | **Inter-User** | | | | | | | |
|  | Inter-User | 14.48 | 0.63 | **4.33%** | 149.2 | 2.22 | **1.49%** | **2.91%** |
|  | **OVERALL** | - | - | **4.26%** | - | - | **1.49%** | **2.88%** |
|  |  | | | | | | | |
| **PR** | **Intra-Run** | | | | | | | |
|  | User 1 | 14.7 | 0.59 | **4.03%** | 149.31 | 2.45 | **1.50%** | **2.77%** |
|  | User 2 | 14.61 | 0.59 | **4.05%** | 149.4 | 2.28 | **1.53%** | **2.79%** |
|  | Cumulative | 14.65 | 0.59 | **4.04%** | 149.35 | 2.36 | **1.52%** | **2.78%** |
|  | **Inter-Run** | | | | | | | |
|  | User 1 | 14.77 | 0.59 | **3.99%** | 149.34 | 2.26 | **1.51%** | **2.75%** |
|  | User 2 | 14.61 | 0.59 | **4.00%** | 149.4 | 2.28 | **1.53%** | **2.77%** |
|  | Cumulative | 14.69 | 0.59 | **4.00%** | 149.37 | 2.27 | **1.52%** | **2.76%** |
|  | **Inter-User** | | | | | | | |
|  | Inter-User | 14.69 | 0.59 | **4.04%** | 149.36 | 2.26 | **1.51%** | **2.78%** |
|  | **OVERALL** | - | - | **4.02%** | - | - | **1.52%** | **2.77%** |
|  |  | | | | | | | |
| **HER2** | **Intra-Run** | | | | | | | |
|  | User 1 | 14.76 | 0.57 | **3.86%** | 149.54 | 2.27 | **1.52%** | **2.69%** |
|  | User 2 | 14.56 | 0.52 | **3.59%** | 149.4 | 2.28 | **1.53%** | **2.56%** |
|  | Cumulative | 14.66 | 0.54 | **3.73%** | 149.47 | 2.27 | **1.53%** | **2.63%** |
|  | **Inter-Run** | | | | | | | |
|  | User 1 | 14.77 | 0.57 | **3.84%** | 149.53 | 2.26 | **1.51%** | **2.68%** |
|  | User 2 | 14.74 | 0.55 | **3.70%** | 149.4 | 2.28 | **1.53%** | **2.62%** |
|  | Cumulative | 14.75 | 0.56 | **3.77%** | 149.46 | 2.27 | **1.52%** | **2.65%** |
|  | **Inter-User** | | | | | | | |
|  | Inter-User | 14.66 | 0.55 | **3.73%** | 149.47 | 2.27 | **1.52%** | **2.63%** |
|  | **OVERALL** | - | - | **3.75%** | - | - | **1.52%** | **2.64%** |
|  |  | | | | | | | |
