# Supplementary material for: Circulating tumor cell assay to non-invasively evaluate PD-L1 and other therapeutic targets in multiple cancers
Source: PLoS One. 2022 Jun 17;17(6):e0270139. doi: 10.1371/journal.pone.0270139 (PMC9205490; doi:10.1371/journal.pone.0270139)
Supplement: S13 Table — (DOCX) [file pone.0270139.s018.docx]

**Analytical Validation - FISH**

*Hybridization Efficiency:*

Hybridization efficiency was established using PMBCs (n= 20) and SKBR3 (n = 5) samples that were processed as described above and enumerating the proportion of cells with positive signal for ERBB2/CEN17 among all nucleated (DAPI+) cells. The overall median hybridization efficiency was determined to be 99.7% (S13 Table).

**S13 Table. Hybridization Efficiency (HER2-FISH analysis)**

| **Sample Type** | **No. of Samples** | **Efficiency** |
| --- | --- | --- |
| PBMC | 20 | 99.9% (87.2% - 100%) |
| SKBR3 | 5 | 99.7% (99.7% - 100%) |
| Overall | 25 | 99.7% (87.2% - 100%) |
| *Median and Range | | |
