# Supplementary material for: Circulating tumor cell assay to non-invasively evaluate PD-L1 and other therapeutic targets in multiple cancers
Source: PLoS One. 2022 Jun 17;17(6):e0270139. doi: 10.1371/journal.pone.0270139 (PMC9205490; doi:10.1371/journal.pone.0270139)
Supplement: S14 Table — (DOCX) [file pone.0270139.s019.docx]

**Analytical Validation - FISH**

*Precision*

Inter-user variability was determined for PMBC samples processed for determination of ERBB2/CEN17 positive cells. Median CV for inter-user variability was 1.2% indicating high precision of the test (S14 Table).

**S14 Table. Precision (HER2-FISH analysis)**

| **Sample Type** | **No of Samples** | **%CV*** |
| --- | --- | --- |
| PBMC | 5 | 1.2% (0.5% - 6.3%) |
| *Median and Range | | |

Precision of the test was defined as the closeness of multiple observations when performed under the same conditions, by same or different operators, on the same or different days, using the same or different instruments. Precision was established by showing a low coefficient of variation (CV, %) between multiple replicates of the same sample evaluated by the same / different operator on the same or different days, using the same or different instruments. For precision, mean and standard deviation (SD) of multiple measurements was obtained by standard formulae in MS Excel.

Mean = (Sum of Observation) / {Total numbers of Observations}

SD= √ *Σ* ((X-x)2/n-1), where X is the value in data distribution, x is the sample mean, n = number of observations.

The mean and SD were used to derive CV = (SD / Mean) × 100 (%)

%CV was also estimated for interference and robustness.
