# Supplementary material for: Circulating tumor cell assay to non-invasively evaluate PD-L1 and other therapeutic targets in multiple cancers
Source: PLoS One. 2022 Jun 17;17(6):e0270139. doi: 10.1371/journal.pone.0270139 (PMC9205490; doi:10.1371/journal.pone.0270139)
Supplement: S15 Table — (DOCX) [file pone.0270139.s020.docx]

**Analytical Validation - FISH**

*Analytical sensitivity:*

Analytical sensitivity was defined as the ability of the test to detect cells with gain of ERBB2 copy where these are known to exist and was established using SKBR3 cells. Median Sensitivity of the test across 5 replicates was 99.7% (S15 Table).

**S15 Table. Analytical Sensitivity (HER2-FISH analysis)**

| **Sample Type** | **No of Samples** | **Sensitivity** |
| --- | --- | --- |
| SKBR3 | 5 | 99.7% (99.3% - 99.7%) |
| *Median and Range | | |
