# Supplementary material for: Circulating tumor cell assay to non-invasively evaluate PD-L1 and other therapeutic targets in multiple cancers
Source: PLoS One. 2022 Jun 17;17(6):e0270139. doi: 10.1371/journal.pone.0270139 (PMC9205490; doi:10.1371/journal.pone.0270139)
Supplement: S16 Table — (DOCX) [file pone.0270139.s021.docx]

**Analytical Validation - FISH**

*Analytical Specificity:*

Analytical specificity was defined as the ability of the test to not detect cells with gain of ERBB2 copy where these are known to be absent and was established using healthy donor PBMCs. Median Specificity of the test across 20 replicates was 100% (S16 Table).

**S16 Table. Analytical Specificity (HER2-FISH analysis)**

| **Sample Type** | **No of Samples** | **Specificity** |
| --- | --- | --- |
| PBMCs | 20 | 100% (95.3% - 100%) |
| *Median and Range | | |
